# Steroid receptor coactivator 3 is a key modulator of regulatory T cell–mediated tumor evasion

**DOI:** 10.1073/pnas.2221707120

**Published:** 2023-05-30

**Authors:** Sang Jun Han, Prashi Jain, Yosef Gilad, Yan Xia, Nuri Sung, Mi Jin Park, Adam M. Dean, Rainer B. Lanz, Jianming Xu, Clifford C. Dacso, David M. Lonard, Bert W. O'Malley

**Affiliations:** ^a^Department of Molecular Cellular Biology, Baylor College of Medicine, Houston, TX 77030; ^b^Nuclear Receptor, Transcription and Chromatin Biology Program, Dan L. Duncan Cancer Center, Baylor College of Medicine, Houston, TX 77030; ^c^Department of Medicine, Baylor College of Medicine, Houston, TX 77030

**Keywords:** steroid receptor coactivator 3, regulatory T cells, adoptive cell transfer, syngeneic murine model of breast cancer, interferon-γ

## Abstract

Steroid receptor coactivator 3 (SRC-3) is most strongly expressed in regulatory T cells (Tregs) and B cells, suggesting that it plays an important role in the regulation of Treg function. Using an aggressive E0771 mouse breast cell line syngeneic immune-intact murine model, we observed that breast tumors were “permanently eradicated” in a genetically engineered tamoxifen-inducible Treg-cell-specific SRC-3 knockout (KO) female mouse that does not possess a systemic autoimmune pathological phenotype. A similar eradication of tumor was noted in a syngeneic model of prostate cancer. A subsequent injection of additional E0771 cancer cells into these mice showed continued resistance to tumor development without the need for tamoxifen induction to produce additional SRC-3 KO Tregs. SRC-3 KO Tregs were highly proliferative and preferentially infiltrated into breast tumors by activating the chemokine (C-C motif) ligand (Ccl) 19/Ccl21/chemokine (C-C motif) receptor (Ccr)7 signaling axis, generating antitumor immunity by enhancing the interferon-γ/C-X-C motif chemokine ligand (Cxcl) 9 signaling axis to facilitate the entrance and function of effector T cells and natural killer cells. SRC-3 KO Tregs also show a dominant effect by blocking the immune suppressive function of WT Tregs. Importantly, a single adoptive transfer of SRC-3 KO Tregs into wild-type E0771 tumor-bearing mice can completely abolish preestablished breast tumors by generating potent antitumor immunity with a durable effect that prevents tumor reoccurrence. Therefore, treatment with SRC-3-deleted Tregs represents an approach to completely block tumor growth and recurrence without the autoimmune side effects that typically accompany immune checkpoint modulators.

## Significance

Tregs are essential in restraining immune responses for immune homeostasis. SRC-3 (Steroid receptor coactivator 3) is a pleiotropic coactivator, the second most highly expressed transcriptional coactivator in Tregs, and a suspect in Treg function. The disruption of SRC-3 expression in Tregs leads to a “complete lifetime eradication” of tumors in aggressive syngeneic breast cancer mouse models because deletion of SRC-3 alters the expression of a wide range of key genes involved in efferent and afferent Treg signaling. SRC-3KO Tregs confer this long-lasting protection against cancer recurrence in mice without an apparent systemic autoimmune pathological phenotype. Therefore, treatment with SRC-3-deleted Tregs could represent an efficient future target for eliminating tumor growth and recurrence without the autoimmune side effects that typically accompany immune checkpoint modulators.

SRC-3 (Steroid receptor coactivator 3) is a pleiotropic coactivator that interacts with a wide range of transcription factors. Cistromic studies of SRC-3 support its pan-genomic transcriptional activities, showing that it is associated with *cis*-regulatory elements across the genome (1). Considering SRC-3’s broad control of gene expression, it is important to consider the mechanistic consequences that are likely to ensue when SRC-3 expression is disrupted. Central to this concept, it is the expectation that perturbation of SRC-3 should result in broad, systemic effects in gene expression and not changes in only a few genes or pathways. Previous studies have shown that whole-body SRC-3 knockout (KO) mice show increased B and T cell lymphoproliferation (2). SRC-3 also has a critical role in generating a tumor-promoting immune microenvironment that enhances breast tumor progression in immune-intact mice (3). For example, SRC-3 inhibition with an SRC-3 small molecular inhibitor (SRC-2 small molecular inhibitor-2 or SI-2) and SRC-3 knockdown in breast cancer cells generates a tumor-suppressive immune microenvironment in tumors by increasing the numbers of tumor-infiltrating cytotoxic immune cells (such as CD4^+^ and CD8^+^ T cells and NK (naturalkiller) cells) and Ifng but reducing CD4^+^Foxp3^+^ regulatory T (Treg) cells compared to controls. Furthermore, SRC-3 is expressed strongly in Tregs (4), suggesting that it may function in Treg-mediated immune suppression. Our observations implicate SRC-3 as a broadly acting and goal-oriented pleiotropic regulator of Treg function that can coordinately control the expression of vast numbers of genes in Tregs involved in effecting immune function.

Tregs are essential in restraining immune responses that maintain the immune homeostasis necessary to prevent autoimmune disease. Tregs act by constraining effector immune cell proliferation and function through a number of distinct mechanisms: 1) Tregs secrete cytokines that inactivate effector T cell (CD4+ T and CD8+ T cells)-mediated cytotoxicity (5–7). 2) Tregs produce perforin and granzyme B that can induce apoptosis in cytotoxic effector T cells (8). Furthermore, 3) Tregs inhibit NK cell proliferation and suppress production of interferon-γ (Infg) through their secretion of transforming growth factor-beta (TGF-β) (9). 4) Treg proliferation is strongly induced by exposure to IL-2 secreted by activated T effector cells. These activated and elevated numbers of Tregs produce several anti-inflammatory cytokines (IL-10, IL-35, and TGF-β) that suppress cytotoxic effector immune cells, forming a negative feedback mechanism to limit the immune response (10). Additionally, 5) Tregs interact with monocytes to prevent M1-type macrophage differentiation while enhancing M2-type macrophage differentiation, leading to the production of IL-10 that suppresses effector T cells (11). Notably, the loss of Treg function can result in pathological chronic autoimmune diseases, so a delicate balance must be met to allow the immune system to attack pathogens and cancer cells while also providing an appropriate level of restraint needed to avoid deleterious autoimmune disease (12).

Forkhead box transcription factor 3 (Foxp3) is a principal transcription factor used to define Treg cellular identity (13). While the vast amount of Treg-oriented immunotherapeutic approaches focus on targeting signaling proteins expressed on the cell membrane, a few studies have sought to understand how transcriptional regulation by Foxp3 modulates Treg-mediated immune homeostasis (14). Focusing on transcriptional regulation in Tregs, we have investigated transcriptional programs within the Treg nucleus, recently identifying SRC-3 as a primary regulator of Treg gene expression (4). SRC-3 has been characterized previously as a prominent oncogene that drives somatic cell cancer progression by activating cell-autonomous growth pathways within cancer cells (15). In addition to cancers, we found previously that SRC-3 has a critical role in immune cell function. For example, SRC-3 is the most highly expressed transcriptional coactivator in Tregs (16). Also, as discussed above, SRC-3 KO mice possess an immune phenotype with elevated numbers of B cells and T cells in their lymph nodes, spleen, and bone marrow (2). SRC-3 is highly expressed in both mouse and human Tregs, and inhibition with an SRC-3 small molecular inhibitor (SI-2) inhibits the immune suppressive function of Tregs (4). Therefore, in addition to its function within cancer cells, we suspected that SRC-3 also possesses a critical role within Tregs and may be responsible for driving immune suppressive functions in Tregs that likely enhance tumor progression.

Herein, we sought to understand the specific role of SRC-3 within the Treg cell compartment, focusing on tumor evasion of the immune system. Using genetically engineered Treg-cell-specific SRC-3 KO mice, we show that disruption of Treg SRC-3 expression leads to a complete lifetime eradication of tumors in aggressive syngeneic breast and prostate cancer models. Notably, these SRC-3 KO Tregs still support immune checkpoint functions in healthy tissues in the animal, avoiding the severe inflammatory consequences associated with complete loss of Tregs, such as in FoxP3 mutant *scurfy* mice. Notable changes in the expression of Ifng, Il-10, Il-35, and Tgf-β and membrane checkpoint inhibitors were observed in SRC-3 KO Tregs, suggesting that deletion of SRC-3 alters the expression of a range of key genes involved in efferent and afferent Treg signaling, consistent with its role as a known pleiotropic regulator of many mammalian genes. It markedly alters the tumor-immune microenvironment in a way that supports tumor destruction by an animal’s own inherent immune system.

## Results

### Tumors Are Eradicated in Treg-Cell-Specific SRC-3 KO Mice.

To directly determine the role of SRC-3 in Treg cell function, Treg-cell-specific SRC-3 KO mice (SRC-^d/d^:Foxp3^Cre/YPF^) were generated by crossing floxed SRC-3 (SRC-3^f/f^, mixed background) mice possessing a flox cassette that brackets known key functional exons 11 and 12 of the *Ncoa3*/*SRC-3* gene mice (17) and Foxp3^Cre/YFP^ mice (B6.129(Cg)-*Foxp3^tm4(YFP/icre)Ayr^*/J) expressing active Cre recombinase in Foxp3^+^ T cells. Treg-cell-specific SRC-3 KO (SRC-3^d/d^:Foxp3^Cre/YPF^) were generated by breeding SRC-3^d/d^:Foxp3^Cre/YPF^ female mice with SRC-3^f/f^ (mixed background) male mice. The SRC-3^f/f^ (mixed background) mice were produced by breeding male and female SRC-3^f/f^ (mixed background) mice. The comparative analysis of RNA expression profile in SRC-3 KO Treg versus WT Tregs from spleens of SRC-^d/d^:Foxp3^Cre/YPF^ (mixed background) and SRC-3^f/f^ (mixed background), respectively, revealed that SRC-3 and Foxp3 levels were significantly reduced in SRC-3 KO Tregs compared to WT Tregs (*SI Appendix*, Fig. S1 *A*–*C*) (18). SRC-3 KO broadly downregulated interleukins, multiple key immune checkpoint genes, chemokine receptors, other key surface proteins, and transcription factors that are required for pro-tumor immune activity in Tregs (*SI Appendix*, Fig. S1 *D*–*F* and Tables S1 and S2). Therefore, SRC-3 KO likely would alter the pro-tumor activity of Tregs. To validate our hypothesis, the genetic background of SRC-3^f/f^ mice was moved into the C57BL/6J isogenic line and then crossed with Foxp3-specific tamoxifen-inducible Cre recombinase mice [Foxp3^tm9(EGFP/Cre/ERT2)Ayr^] (Foxp3^Cre-ERT2/+^) to generate bigenic SRC-3^f/f^:Foxp3^Cre-ERT2/+^ mice on a pure C57BL/6J background (*SI Appendix*, Fig. S2*A*). SRC-3^f/f^:Foxp3^Cre-ERT2/+^ and SRC-3^f/f^ littermates were generated by breeding SRC-3^f/f^:Foxp3^Cre-ERT2/+^ female mice (C57BL/6J background) and SRC-3^f/f^ male mice (C57BL/6J background). Therefore, SRC-3^f/f^:Foxp3^Cre-ERT2/+^ and SRC-3^f/f^ female mice used in all subsequent cancer studies have a genetic background of C57BL/6J. After activating the Cre-ERT2 recombinase with tamoxifen in these bigenic SRC-3^f/f^:Foxp3^Cre-ERT2/+^ mice, Treg-cell-specific SRC-3 KO (SRC-3^d/d^:Treg mice) were created (*SI Appendix*, Fig. S2*B*). Genomic PCR and DNA sequencing of the PCR products validated the deletion of critical functional exons 11 and 12 of the SRC-3 gene in Tregs in spleens of SRC-3^f/f^:Foxp3^Cre-ERT2/+^ female mice (*SI Appendix*, Fig. S2 *C* and *D*) (17). Double immunofluorescent staining with antibodies against SRC-3 and Foxp3 in spleens revealed that 65.2% of Foxp3^+^ cells were Foxp3^+^SRC-3^+^ in the spleen of SRC-3^f/f^ female mice, but only 12.1% of Foxp3^+^ cells were Foxp3^+^SRC-3^+^ cells in the spleens of SRC-3^d/d^Treg mice (*SI Appendix*, Fig. S3*A*). Therefore, SRC-3 expression was lost in 53.1% of Foxp3^+^ cells in the spleens of SRC-3^d/d^:Treg mice. For further validation of SRC-3 KO in Tregs, Tregs and Tconv were isolated from the spleens of SRC-3^f/f^ and SRC-3^d/d^:Treg female mice using the Magnetic-Activated Cell Sorting (MACS) Column–based CD4^+^CD25^+^Regulatory T Cell Isolation Kit and then analyzed by flow cytometry with an antibody against CD25 and Foxp3. (*SI Appendix*, Fig. S3*B*). The percentage of CD25^+^Foxp3^+^ Treg in WT and SRC-3 KO Tregs from the spleens was 80.0 and 92.9%, respectively. However, the percentage of CD25^+^Foxp3^+^ Tregs counted from total T cells from the spleen of SRC-3^d/d^:Treg was only 18.2%. High purities of isolated WT and SRC-3 KO Treg were achieved. Double immunofluorescent staining revealed that 54.1% of Foxp3^+^ cells were Foxp3^+^SRC-3^+^ in CD4^+^CD25^+^ Tregs from SRC-3^f/f^ mice, but only 13.0% of Foxp3^+^ cells were Foxp3^+^SRC-3^+^ in CD4^+^CD25^+^ Tregs from SRC-3^d/d^:Treg mice (*SI Appendix*, Fig. S3*C*). Also, a comparison of SRC-3 mRNA levels in Tregs from the spleens of SRC-3^d/d^:Treg female mice with their SRC-3^f/f^ controls revealed a significant reduction in expression (*SI Appendix*, Fig. S3*D*). Collectively, these data show that SRC-3 expression is strongly abrogated in Tregs of SRC-3^d/d^:Treg female mice.

Body weights of SRC-3^d/d^:Treg female mice over their lifetime were not statistically different from those of SRC-3^f/f^ female mice (*SI Appendix*, Fig. S4*A*). Reproductive capacity is a hallmark of mouse health. Notably, mouse fertility analysis revealed that the number of pups produced by SRC-3^d/d^:Treg female mice mated with wild-type male mice was not statistically different from that produced by SRC-3^f/f^ female mice mated with wild-type male mice (*SI Appendix*, Fig. S4*B*). *Foxp3*^−/−^ *scurfy* mice possess a severe, systemic autoimmune phenotype and die at an early age (19, 20). Unlike *scurfy* mice, however, SRC-3^d/d^:Treg female mice have similar levels of blood cytokine/chemokines compared to SRC-3^f/f^ female mice, with only three observable cytokines (Timp-1, Ccl2, and Il-1ra) elevated in the SRC-3^d/d^:Treg mice (*SI Appendix*, Fig. S4 *C* and *D*). In addition to their cytokine profiles, immuno-phenotyping analysis of the spleens of SRC-3^d/d^:Treg mice did not show an altered repertoire of lymphocytes and myeloid cells compared to that of SRC-3^f/f^ female mice (*SI Appendix*, Fig. S4 *E* and *F*). These data indicate that SRC-3^d/d^:Treg mice do not possess the pathological characteristics observed in mice with entirely abrogated Treg function or with checkpoint inhibitor agents.

**Fig. 1.**
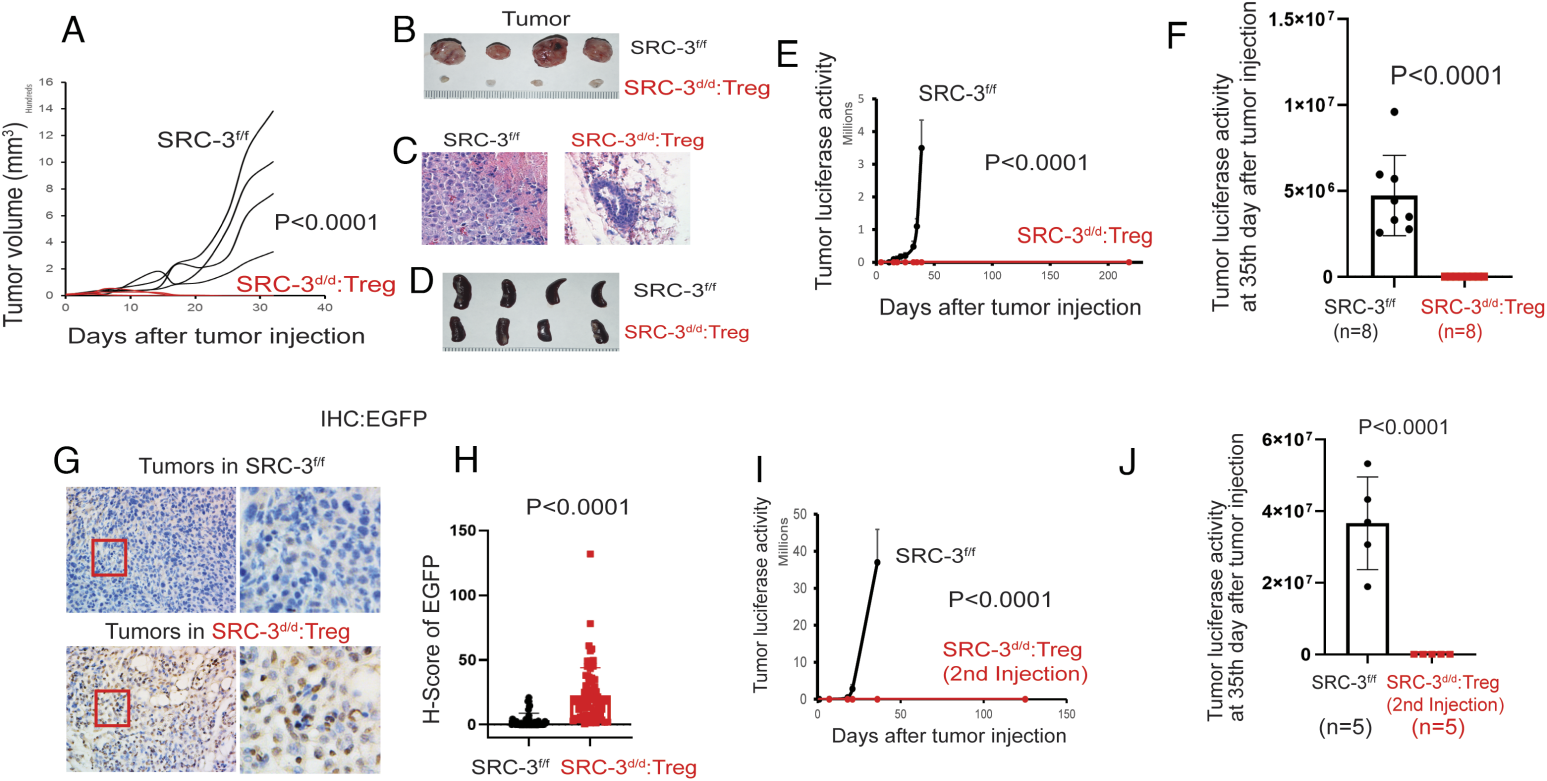
E0771 breast tumor eradication in SRC-3^d/d^:Treg mice. (*A*) E0771 breast tumor growth in SRC-3^f/f^ versus SRC-3^d/d^:Teg female mice. (*B*) Breast tumors isolated from SRC-3^f/f^ and SRC-3^d/d^:Teg female mice on the 33rd day after injection with E0771 cells. (*C*) H&E staining of tumors is presented in panel *B*. (*D*) Spleens isolated from SRC-3^f/f^ and SRC-3^d/d^:Teg female mice on the 40th day after tamoxifen treatment. (*E*) Breast tumor luciferase activity in SRC-3^f/f^ and SRC-3^d/d^:Treg female mice. (*F*) The repeated tumor luciferase activity experiment with tamoxifen-treated SRC-3^f/f^ (n = 8) and SRC-3^d/d^:Treg mice (n = 8) was determined on the 35th day after E0771:LUC cell injection. (*G*) IHC analysis of EGFP+ cells in tumors of tamoxifen-treated SRC-3^f/f^ and SRC-3^d/d^:Treg mice on the 14th day after E0771 cell injection. (*H*) H-score for EGFP+ cells in tumors presented in panel *G*. (*I*) Tumor luciferase activity in SRC-3^f/f^ and breast cancer–eradicated SRC-3^d/d^:Treg mice after a 2nd injection of E0771:LUC cells. (*J*) The repeated experiment for tumor luciferase activities in tamoxifen-treated SRC-3^f/f^ (n = 5) and breast tumor–eradicated SRC-3^d/d^:Treg mice (n = 5) on the 35th day after E0771:LUC cell reinjection.

E0771 cells, an aggressive breast cancer cell line established from a spontaneous mammary gland carcinoma in the C57BL/6 mouse strain, were used to characterize how SRC-3 KO Tregs impact breast cancer progression in the immune-intact mouse (21). E0771 cells were orthotopically injected into the mammary fat pads of SRC-3^d/d^:Treg and SRC-3^f/f^ female mice (*SI Appendix*, Fig. S5*A*). E0771 breast tumors grew aggressively in SRC-3^f/f^ female mice (Fig. 1 *A* and *B*). Early E0771 breast tumors were detected in SRC-3^d/d^:Treg female mice 5 d after E0771 cell injection, but breast tumors became undetectable after 20 d (Fig. 1 *A* and *B*). Hematoxylin and eosin (H&E) analyses validated eradication of the primary breast tumors in SRC-3^d/d^:Treg female mice, and no tumor masses were detected in mammary fat pads of these animals (Fig. 1*C*). Also, SRC-3^d/d^:Treg female mice did not show splenomegaly, with spleens appearing similar to SRC-3^f/f^ female control mice (Fig. 1*D*). In Foxp3^Cre-ERT2/+^ monogenic mouse lines, E0771 breast tumors also grew aggressively (*SI Appendix*, Fig. S6 *A*–*D*). These observations confirm that the lack of tumor growth in the SRC-3^f/f^:Foxp3^Cre-ERT2/+^ bigenic mice was not due to mouse background genetic issues. To rule out the potential direct effects of tamoxifen on breast tumors, tumor-bearing SRC-3^f/f^ female mice were treated with tamoxifen versus vehicle (*SI Appendix*, Fig. S7*A*). Tumor luciferase activity analysis revealed that tamoxifen treatment did not impact the growth of E0771 breast tumors in SRC-3^f/f^ female control mice (*SI Appendix*, Fig. S7 *B* and *C*).

To noninvasively analyze tumor progression, stable luciferase-expressing E0771 (E0771:LUC) cells were developed by infection with a lentiviral luciferase expression vector. To determine whether one injection of SRC-3 KO Tregs can prevent tumor initiation, E0771:LUC cells were injected into SRC-3^f/f^:Foxp3^Cre-ERT2/+^ female mice after first treating them with tamoxifen or vehicle control (*SI Appendix*, Fig. S8*A*). E0771 breast tumors developed aggressively in SRC-3^f/f^:Foxp3^Cre-ERT2/+^ female mice treated with vehicle, but tumors failed to progress beyond a short initial period of growth in SRC-3^f/f^:Foxp3^Cre-ERT2/+^ female mice treated with tamoxifen (*SI Appendix*, Fig. S8 *B* and *C*). Next, we sought to examine further whether SRC-3 KO Tregs can drive the regression of “preexisting” E0771 tumors in mice. SRC-3^f/f^:Foxp3^Cre-ERT2/+^ and SRC-3^f/f^ female mice were first injected with E0771:LUC breast cancer cells. After tumors were allowed to grow for 7 d, mice were treated with tamoxifen (*SI Appendix*, Fig. S8*D*). Tumors again were eradicated in SRC-3^f/f^:Foxp3^Cre-ERT2/+^ mice that were treated with tamoxifen. In contrast, tumors continued to progress in tamoxifen-treated SRC-3^f/f^ female mice (*SI Appendix*, Fig. S8 *E* and *F*). Compared to tamoxifen treatment, breast tumors grew aggressively in SRC-3^f/f^:Foxp3^Cre-ERT2/+^ female mice treated with vehicle (*SI Appendix*, Fig. S8 *G*–*I*). Therefore, SRC-3 KO Tregs induced with tamoxifen treatment eradicate breast tumors in SRC-3^d/d^:Treg female mice.

The biggest problem in current cancer therapies is tumor recurrence after cessation of treatment. Thus, breast tumor recurrence in SRC-3^d/d^:Treg female mice was examined. E0771:LUC cells were orthotopically injected into SRC-3^f/f^ and SRC-3^d/d^:Treg female mice. E0771 breast tumors became undetectable in SRC-3^d/d^:Treg female mice 20 d after E0771:Luc cell injection, and tumor recurrence was not detected in SRC-3^d/d^:Treg female mice for >218 d after E0771:LUC cell injection (Fig. 1*E* and *SI Appendix*, Fig. S9 *A* and *B*); we consider these tumors to be permanently eliminated from the SRC-3^d/d^:Treg female mice. We repeated this experiment with increased animal numbers, confirming the complete eradication of tumors in SRC-3^d/d^:Treg female mice (n = 8) but not in SRC-3^f/f^ female mice (n = 8) (Fig. 1*F*).

The SRC-3 KO Tregs express enhanced-Enhanced Green Fluorescent Protein (EGFP) as a result of the bigenic animals possessing the Foxp3^tm9(EGFP/Cre/ERT2)Ayr^ cassette (*SI Appendix*, Fig. S2*B*). Tumors were isolated from SRC-3^d/d^:Treg and SRC-3^f/f^ female mice 14 d after E0771 cell injection (at an early time point when tumors were not yet entirely eradicated in SRC-3^d/d^:Treg female mice). IHC (immunohistochemical) analysis with an EGFP antibody revealed the presence of large numbers of EGFP+ Tregs within the tumors of SRC-3^d/d^:Treg female mice but not in tumors of SRC-3^f/f^ control mice (Fig. 1 *G* and *H*). These results indicate that SRC-3 KO Tregs infiltrate into E0771 tumors before tumor eradication in the SRC-3^d/d^:Treg mice.

The above observation raised the question of whether another type of cancer can be eradicated in the SRC-3^d/d^:Treg mouse model. To address this question, the RM-1 syngeneic immune-intact mouse model of prostate cancer was employed (22). Orthotopically injected luciferase-labeled RM-1 prostate cancer cells developed into prostate cancers in SRC-3^f/f^ male mice but not in SRC-3^d/d^:Treg male mice (*SI Appendix*, Fig. S10*A*). Furthermore, after prostate tumors disappeared, prostate cancer recurrence was not detected in the SRC-3^d/d^:Treg males extending to at least >150 d (*SI Appendix*, Fig. S10*B*). Thus, in a similar fashion to that observed in the E0771 breast cancer model, we found that SRC-3 KO Tregs also can eliminate prostate tumors in an aggressive male prostate cancer mouse model.

The lack of E0771 tumor recurrence in SRC-3^d/d^:Treg mice raised the question of whether long-term resistance to subsequent new tumor formation would occur. To address this question, E0771:LUC cells were reinjected into tumor-eradicated SRC-3^d/d^:Treg mice (*SI Appendix*, Fig. S5*B*) and SRC-3^f/f^ female mice as controls. Tumors grew rapidly in SRC-3^f/f^ control female mice, but “reinjected” E0771 cells could not establish breast tumors in tumor-eradicated SRC-3^d/d^:Treg female mice (at least >125 d) (Fig. 1*I* and *SI Appendix*, Fig. S9*C*). A repeated experiment with an increased number of animals confirmed the tumor resistance phenotype in all SRC-3^d/d^:Treg female mice (n = 5) (Fig. 1*J*).

**Fig. 2.**
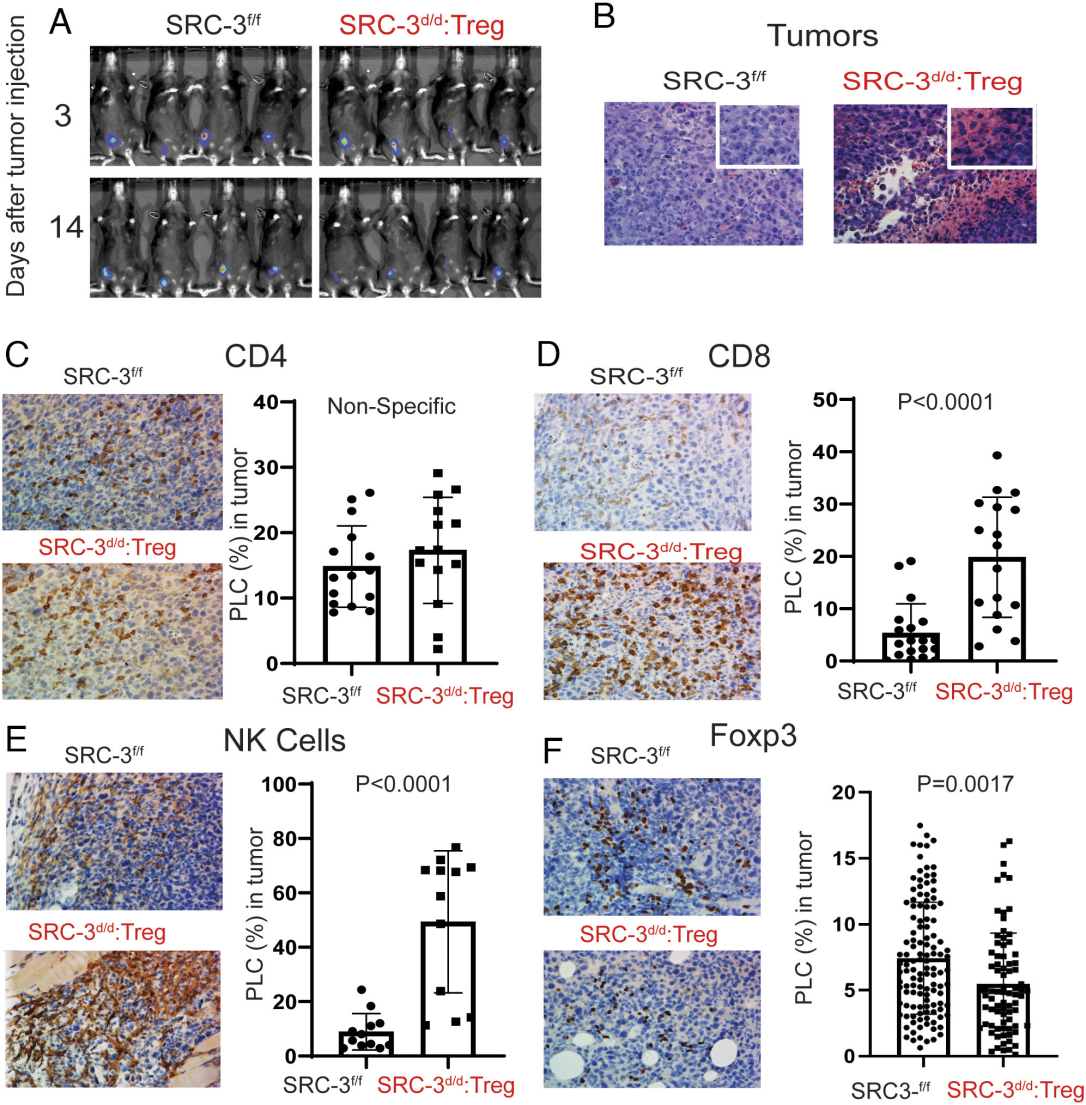
SRC-3^d/d^:Treg mice generate a tumor-suppressive immune microenvironment in breast tumors. (*A*) Images of tumor luciferase activity in SRC-3^f/f^ and SRC-3^d/d^:Treg mice on the 3rd and 14th day after E0771:LUC cell injection. (*B*) H&E staining of tumors in SRC-3^f/f^ and SRC-3^d/d^:Treg mice on the 14th day after E0771:LUC cell injection. (*C*–*F*) IHC analysis of CD4^+^ T cells (*C*), CD8^+^ T cells (*D*), CD49b^+^ NK cells (*E*), and Foxp3+ Tregs (*F*) in tumors from SRC-3^f/f^ and SRC-3^d/d^:Treg mice on the 14th day after E0771:LUC cell injection. The QuPath program was used to quantify IHC staining results. PLC, percentage of labeled cells.

Next, we wished to determine how SRC-3 KO Tregs support tumor eradication at a cell mechanistic level with a focus on how these cells modulate other immune cells in the tumor microenvironment. Since E0771 breast tumors were still present and available for analysis in SRC-3^d/d^:Treg female mice until 14 d after E0771 cell injection (Fig. 2*A*), tumors were isolated from these mice and SRC-3^f/f^ control mice at this time. H&E staining revealed extensive hypocellularity in tumors from the SRC-3^d/d^:Treg mice but not in SRC-3^f/f^ mice (Fig. 2*B*). Tregs usually suppress and inactivate effector immune cells including CD4^+^ T, CD8^+^ T, and NK cells in the tumor immune microenvironment, facilitating tumor progression. The numbers of infiltrating CD4^+^ T cells in tumors from SRC-3^d/d^:Treg mice when compared to that from SRC-3^f/f^ mice (Fig. 2*C*), CD8^+^ T cells and CD49b^+^ NK cells were significantly elevated in tumors from SRC-3^d/d^:Treg female mice when compared to SRC-3^f/f^ mice (Fig. 2 *D* and *E*). Foxp3 immunostaining revealed fewer Foxp3+ Tregs in tumors from SRC-3^d/d^:Treg mice compared to SRC-3^f/f^ control mice (Fig. 2*F*). Therefore, the markedly increased number of infiltrating CD8^+^ and NK effector immune cells observed in tumors from the SRC-3^d/d^:Treg mice was consistent with establishment of an antitumor immune microenvironment by the SRC-3 KO Tregs.

**Fig. 3.**
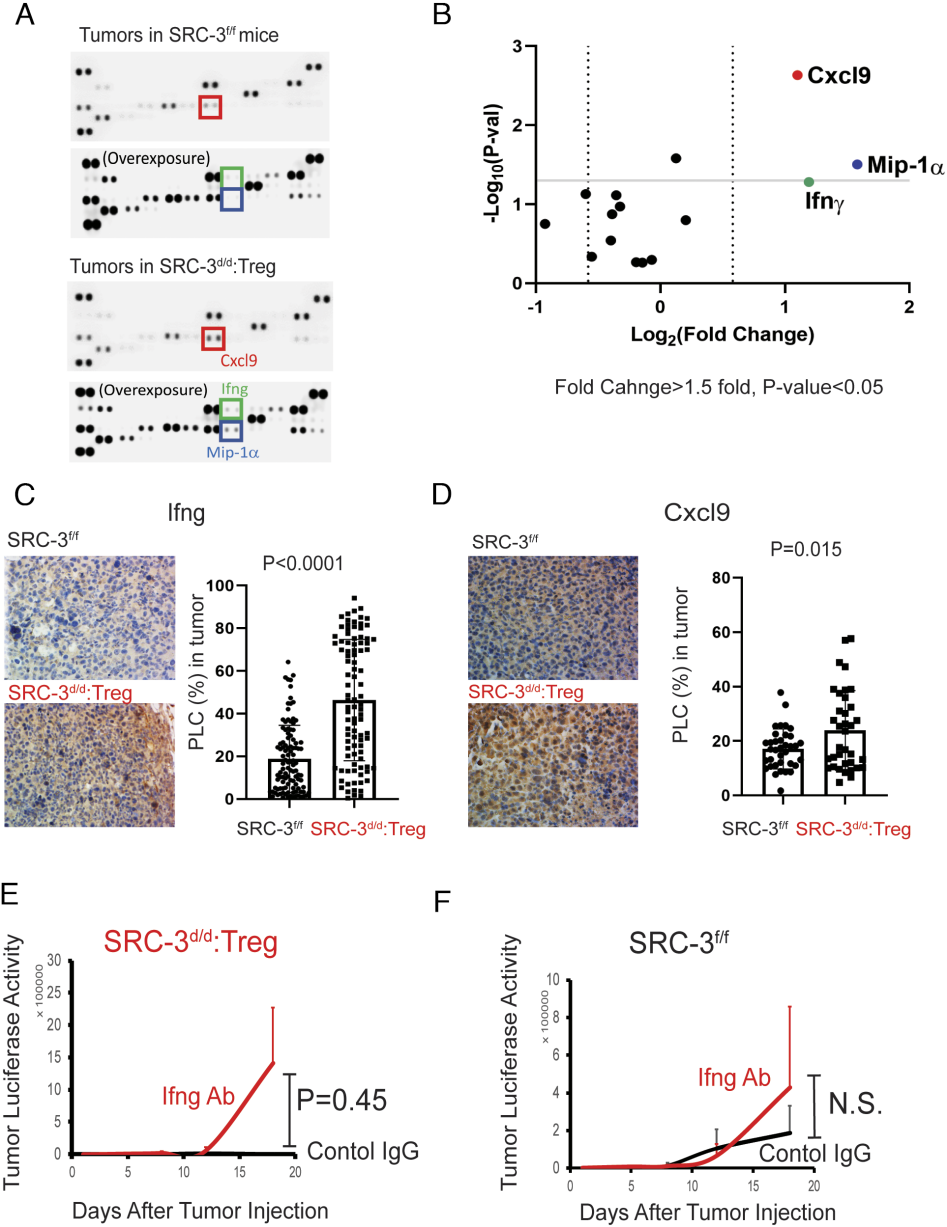
SRC-3^d/d^:Treg mice possess an elevated Ifng/Cxcl9 signaling axis in breast tumors. (*A*) Cytokine/chemokine profiling in breast tumors obtained from SRC-3^f/f^ and SRC-3^d/d^:Treg female mice on the 14th day after E0771:LUC cell injection. (*B*) Quantification of cytokines and chemokines shown in panel *A*. (*C* and *D*) IHC analysis of Ifng (*C*) and Cxcl9 (*D*) in tumors from SRC-3^f/f^ and SRC-3^d/d^:Treg mice on the 14th day after E0771:LUC breast cancer cell injection using the QuPath program. (*E* and *F*) Tumor luciferase activity of tumor-bearing SRC-3^d/d^:Treg (*E*) and SRC-3^f/f^ (*F*) female mice treated with anti-Ifng antibody (Ifng Ab) and control Rat IgG (Control IgG). PLC, percentage of labeled cells.

The cytokine/chemokine milieu within tumors alters the tumor immune microenvironment and strongly impacts cancer progression (23). To investigate the consequences of SRC-3 KO Treg infiltration into E0771 tumors, we performed cytokine/chemokine profiling from the tumors of SRC-3^d/d^:Treg and SRC-3^f/f^ mice 14 d after E0771:LUC cell injection; we observed that levels of Ifng, Cxcl (C-X-C motif chemokine ligand) 9, and Mip-1α are significantly elevated in tumors of SRC-3^d/d^:Treg female mice compared to controls (Fig. 3 *A* and *B*). In addition, IHC analysis validated marked increases in Ifng and Cxcl9 levels in tumors from SRC-3^d/d^:Treg compared to SRC-3^f/f^ control mice (Fig. 3 *C* and *D*). To determine whether Ifng is a critical factor in breast cancer eradication by SRC-3 KO Tregs, breast tumor–bearing SRC-3^f/f^ and SRC-3^d/d^:Treg female mice were intraperitoneally injected with anti-Ifng antibody and rat IgG as the control IgG (*SI Appendix*, Fig. S11*A*). The Ifng depletion by anti-Ifng antibody reactivated E0771 breast tumor progression in SRC-3^d/d^:Treg female mice compared to control IgG (Fig. 3*E* and *SI Appendix*, Fig. S11*B*). However, anti-Ifng antibody treatment did not impact the breast tumor growth in SRC-3^f/f^ female mice (Fig. 3*F* and *SI Appendix*, Fig. S11*B*). Therefore, Ifng appears to be essential for breast tumor eradication in SRC-3^d/d^:Treg female mice. Ifng is a central proinflammatory cytokine that can suppress tumor progression and activate CD8^+^ T cells and NK cells (24, 25). Cxcl9 is a chemokine that recruits and activates Cxcr3^+^ immune cells, including CD8^+^ and NK cells (26). We conclude that SRC-3 KO Tregs strongly enhance the Ifng/Cxcl9 axis in E0771 tumors to support the establishment of an antitumor immune microenvironment by recruiting and activating CD8^+^ and NK effector immune cells. In contrast to its large increase in the tumor microenvironment, Ifng was not systemically elevated in serum from these animals (*SI Appendix*, Fig. S4*C*).

### SRC3 KO Tregs Possess a “Functional” Dominant Role in Tumor Immunity.

**Fig. 4.**
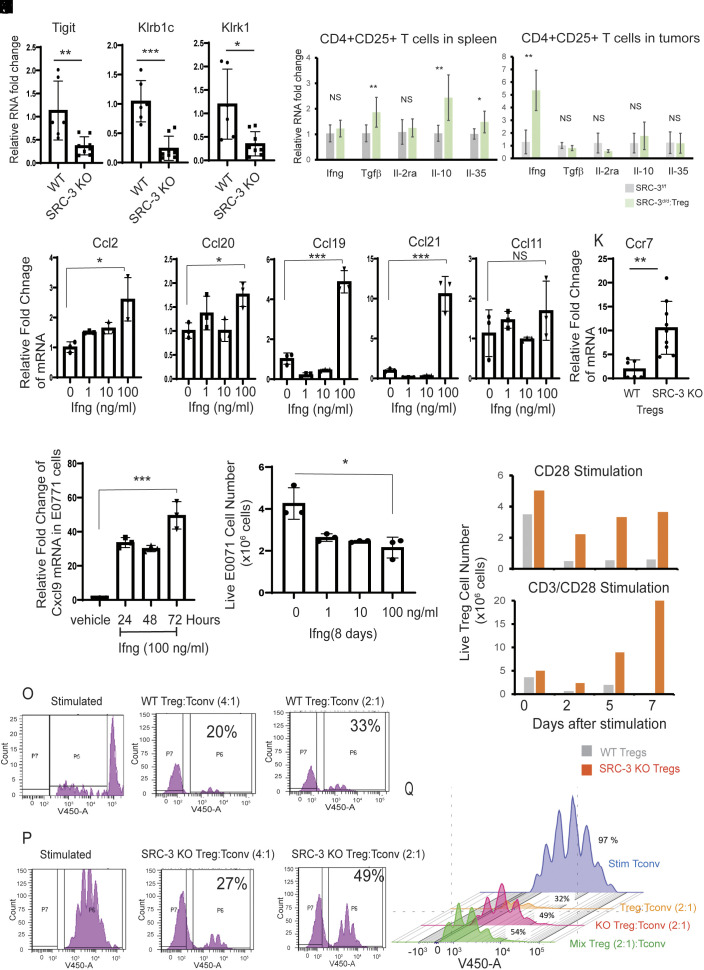
Molecular properties of SRC-3 KO Treg cells. (*A*–*C*) Relative RNA levels of Tigit (*A*), Klrb1c (*B*), and Klrk1 (*C*) in WT versus SRC-3 KO Treg cells from spleens. (*D* and *E*) Relative mRNA levels for Ifng, Tgf-β, Il-2ra, Il-10, and Il-35 in CD4^+^CD25^+^ Treg cells isolated from spleens (*D*) and breast tumors (*E*) in breast tumor–bearing SRC-3^f/f^ and SRC-3^d/d^:Treg mice on the 14th day after E0771:LUC cell injection. (*F*–*J*) Relative RNA levels of Ccl2 (*F*), Ccl20 (*G*), Ccl19(*H*), Ccl21(*I*), and Ccl11(*J*) in E0771 breast cancer cells treated with 0, 1, 10, and 100 ng/mL of Ifng. (*K*) RNA levels of Ccr7 in WT versus and SRC-3 KO Tregs. (*L*) RNA analysis of Cxcl9 in E0771 breast cancer cells upon Ifng (100 ng/mL) treatment for 24, 48, and 72 h. (*M*) The viability of E7001 breast cell numbers upon 1, 10, and 100 ng/mL of Ifng for 8 d. (*N*) The viability of WT Tregs and SRC-3 KO Tregs at 0, 2, 5, and 7th days after CD28 and CD3/CD28 stimulation. (*O*) Flow cytometry analysis of T cells showing immune suppressive activity of WT Tregs. (*P*) Flow cytometry analysis of T cells showing the reduced immune suppressive activity of SRC-3 KO Tregs. (*Q*) The reduced suppressive activity of the mixture of SRC-3 KO and WT Tregs (1:1) to Tconv cell proliferation when compared to WT Tregs. *P < 0.05; **P < 0.01; ***P < 0.001; NS, nonspecific.

How do SRC-3 KO Tregs eradicate breast tumors even though WT Tregs exist in animals and in breast tumors? Treg RNA analysis revealed the down-regulation of Tigit, Klrb1c, and Klrk1 in SRC-3 KO Tregs compared to WT Tregs (Fig. 4 *A*–*C* and *SI Appendix*, Fig. S1*F*). Since Tigit, Klrb1c, and Klrk1 are coinhibitory molecules in Tregs that inhibit proinflammatory T cell responses selectively (27), the downregulation of these coinhibitory molecules after SRC-3 KO would be expected to disrupt immune suppressive functions of Tregs. Tregs also were isolated from tumors and spleens of tumor-bearing SRC-3^d/d^:Treg and SRC-3^f/f^ mice 14 d after E0771 breast cancer cell injection. RNA analysis by qPCR with Treg cell marker genes revealed that SRC-3 KO Tregs in spleens of tumor-bearing SRC-3^d/d^:Treg female mice had elevated expression of anti-inflammatory cytokines such as Tgf-β, Il-10, and Il-35 compared to Tregs from the spleens of tumor-bearing SRC-3^f/f^ female mice (Fig. 4*D*). In contrast, SRC-3 KO Tregs in tumors from tumor-bearing SRC-3^d/d^:Treg female mice had significantly elevated expression of Ifng (~fivefold) compared with Tregs from the tumors in tumor-bearing SRC-3^f/f^ female mice (Fig. 4*E*). These results reveal that the molecular phenotypes of SRC-3 KO Tregs are very different in spleens versus tumors, and SRC-3 KO Tregs in breast tumors promote the antitumor environment by inducing expression of Ifng and other cytokines. Thus, T cell receptor (TCR) activation appears likely to be a critical event for the induction of Ifng in SRC-3 KO Tregs in breast tumors.

What is the role of Infg in breast tumor eradication by SRC-3 KO Tregs? Ifng treatment significantly increased Ccl19/Ccl21 (Treg chemotactic ligands) in E0771 breast cancer cells in contrast to other Treg chemotactic ligands such as Ccl2, Ccl20, and Ccl 11 (Fig. 4 *F*–*J*). Our RNA-seq analysis revealed that the level of Ccr [chemokine (C-C motif) receptor] 7 (Ccl19/Ccl21 receptor) was significantly elevated in SRC-3 KO Tregs compared to WT Tregs (Fig. 4*K*). Therefore, Ifng activates the Ccl19/Ccl21/Ccr7 axis between breast tumors and SRC-3 KO Tregs to signal the recruitment of SRC-3 KO Tregs into breast tumors. Ifng treatment also significantly increased the level of Cxcl9 mRNA expression in E0771 cells (Fig. 4*L*) and suppressed the viability of E0771 cells compared to vehicle (Fig. 4*M*). Thus, Ifng from SRC-3 KO Tregs elevates Cxcl9 expression in E0771 breast tumors in SRC-3^d/d^:Treg female mice and suppresses the growth of breast tumors. Additional in vitro cell survival analysis with Tregs shows that the viability of WT Treg cells from spleens of SRC-3^f/f^ female mice declined over a 7-d period, but the viability of SRC-3 KO Tregs was not reduced over the same time period (Fig. 4*N*). Also, in the presence of CD3/CD28 costimulation, the viability and proliferation of SRC-3 KO Tregs were significantly elevated during in vivo culture compared to WT Tregs (Fig. 4*N*). These data are consistent with our findings that SRC-3 KO Tregs are hearty cells that “functionally predominate” over WT Tregs in the tumor microenvironment.

Next, we sought to determine whether SRC-3 KO impacts the immune suppressive function of Tregs using coculture assays consisting of CD4^+^ CD25^+^ (Tregs) mixed with conventional CD4^+^ CD25^-^ (Tconv) cells from the spleens of SRC-3^d/d^:Treg and SRC-3^f/f^ female mice. Wild-type Tregs suppressed stimulated Tconv cell proliferation (Fig. 4*O*), while SRC-3 KO Tregs suppressed Tconv cell proliferation to a substantially smaller extent (Fig. 4*P*). Moreover, a 2:1 mixture of wild-type and SRC-3 KO Tregs led to the same attenuated suppression of Tconv cell proliferation (Fig. 4*Q*). Therefore, SRC-3 KO Tregs effectively inhibited the Tconv suppressive activity of WT Tregs. Collectively, the above observations suggest that SRC-3 KO Tregs have a “functionally dominant” role in eradicating breast cancer, irrespective of the suppressive effects of neighboring wild-type Tregs.

### Adoptive Cell Transfer (ACT) with SRC-3 KO Tregs Eliminates E0771 Tumors.

**Fig. 5.**
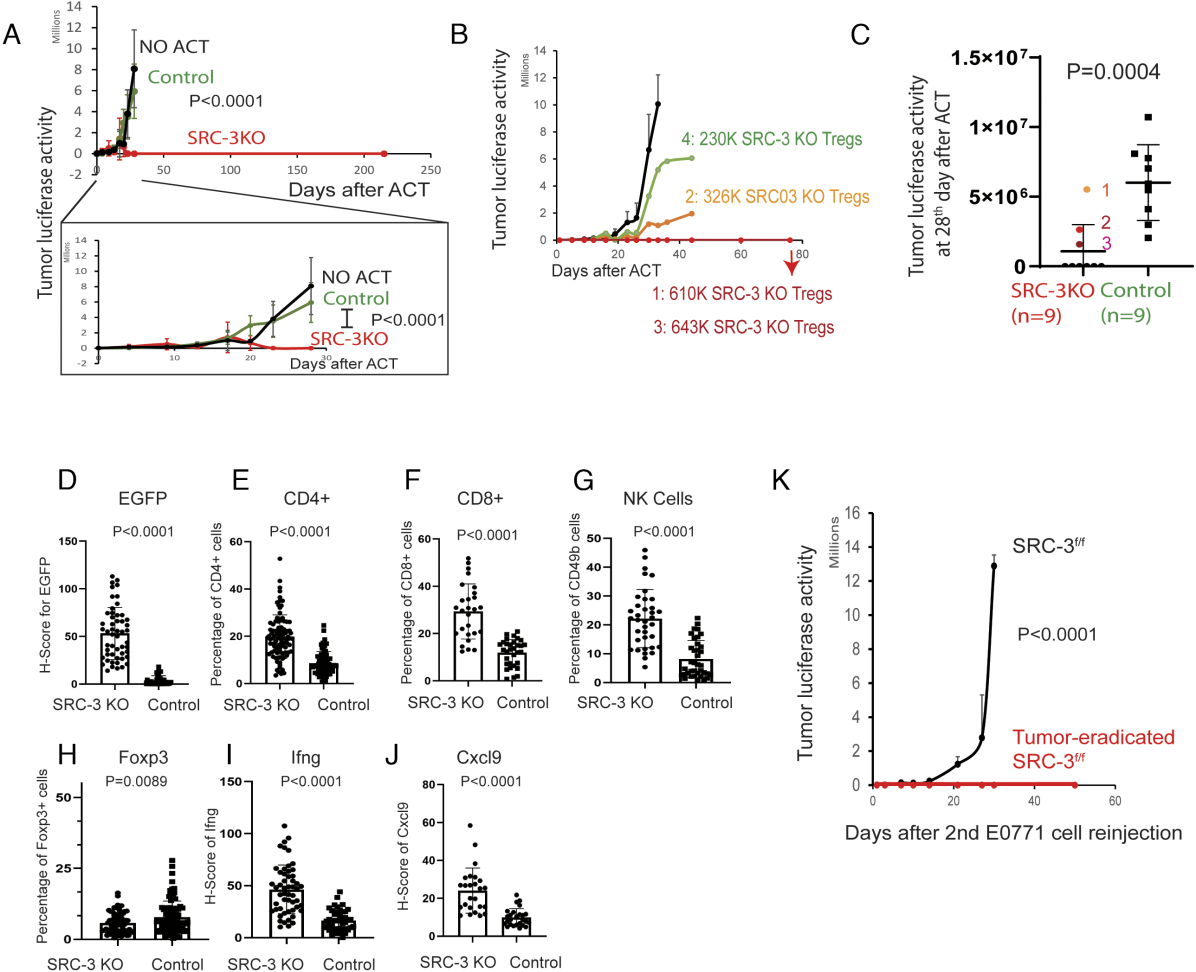
Tumor eradication through adoptive SRC-3 KO Treg transfer. (*A*) Tumor luciferase image analysis in tumor-bearing SRC-3^f/f^ female mice after ACT with SRC-3 KO Tregs (800K cells) and wild-type Tregs (800K cells). Control tumor-bearing SRC-3^f/f^ mice that did not undergo ACT (NO ACT). Tumor luciferase image analysis up to the 30th day, which is in the box, and 215th day after ACT. (*B*) Tumor luciferase activity in SRC-3^f/f^ female mice after ACT with different doses of SRC-3 KO Tregs. Tumor-bearing SRC-3^f/f^ female mice were adoptively transferred with wild-type (800K cells) or SRC-3 KO Treg cells (1: 610K cells, 2: 326K cells, 3: 643K cells, and 4: 230K cells). Afterward, the luciferase activity emanating from tumors was determined. (*C*) Repeated ACT experiment with increased animal numbers. Tumor luciferase activity in SRC-3^f/f^ female mice adoptively transferred with SRC-3 KO Tregs (1: 230K cells, 2: 314K cells, 3: 358K cells, and others: 800K cells, n = 9) and wild-type Tregs (800K cells, n = 9) was determined on the 28th day after E0771:LUC cell injection. (*D*–*J*) IHC analysis of EGFP+ cells (*D*), CD4^+^(*E*), CD8^+^ (*F*), CD49b^+^ (*G*), Foxp3 (*H*), Ifng (*I*), and Cxcl9 (*J*) in breast tumors of female mice at 14th day after ACT with SRC-3KO Tregs (SRC-3 KO) versus WT Tregs (Control). (*K*) Tumor luciferase activity in tumor-eradicated SRC-3^f/f^ female mice after ACT with SRC-3 KO Tregs and SRC-3^f/f^ female control mice after a 2nd injection of E0771:LUC cells.

ACT with Tregs has been promoted as an immunotherapy to treat various chronic autoimmune diseases but not for treating solid cancers (28). Since SRC-3 KO Tregs have a tumor-eradicating activity and a suppressive activity against WT Tregs, we wished to determine whether a “single injection” of ACT with purified SRC-3 KO Tregs would eradicate preexisting E0771 tumors growing in wild-type mice by suppressing the inherent function of WT Tregs. SRC-3 KO and control wild-type Tregs were isolated from the spleens of SRC-3^d/d^:Treg and SRC-3^f/f^ female mice, respectively, and then injected Tregs (800K cells) into tumor-bearing SRC-3^f/f^ female littermates (*SI Appendix*, Fig. S12 *A* and *B*). E0771:LUC cells were developed into breast tumors in SRC-3^f/f^ female mice before conducting ACT (*SI Appendix*, Fig. S13*A*). Notably, ACT with SRC-3 KO Tregs “completely eradicated” established tumors in SRC-3^f/f^ littermates, but ACT with purified wild-type Tregs or no ACT did not suppress breast tumor growth in SRC-3^f/f^ littermates (Fig. 5*A* and *SI Appendix*, Fig. S13*B*). Furthermore, after the initial regression of tumors to undetectable levels through ACT with SRC-3 KO Tregs, tumor recurrence was not observed in these animals for >215 d after ACT (Fig. 5*A*).

We next set up ACT cell dose–response experiments. In instances with a greater number of cells, ACT with SRC-3 KO Tregs (>600K cells) effectively eradicated tumors (Fig. 5*B* and *SI Appendix*, Fig. S13*C*). However, ACT with lower doses of SRC-3 KO Tregs (2: 326K cells and 4: 230K cells) suppressed breast tumor growth compared to ACT with purified wild-type Tregs but did not eradicate breast tumors (Fig. 5*B* and *SI Appendix*, Fig. S13*C*). As expected, ACT with wild-type Tregs (800K cells) failed to block tumor growth (Fig. 5*B* and *SI Appendix*, Fig. S13*C*). The repeat experiment with an increased number of animals (n = 9) also confirmed that the low dose of SRC-3 KO Tregs (1: 230K cells, 3: 314K cells, 3: 358K cells) did not eradicate breast tumors compared to the high dose of SRC-3 KO Tregs (Fig. 5*C*). Therefore, the number (~600K cells) of SRC-3 KO Tregs injected during ACT is a critical factor for achieving complete breast tumor eradication in mice.

Breast tumor masses were still detected in E0771:LUC SRC-3^f/f^ recipient female mice up to 14 d after ACT with SRC-3 KO Tregs (Fig. 5*A*). To examine these tumors before their ultimate clearance, tumors were dissected from SRC-3 KO and WT ACT recipient animals 14 d after ACT cell injection. Because SRC-3 KO Tregs express EGFP, IHC with an EGFP antibody was used to trace the SRC-3 KO Tregs that infiltrated into the tumors of SRC-3^f/f^ host mice. As expected, EGFP expression was detected in the tumors of SRC-3^f/f^ female mice receiving ACT with SRC-3 KO Tregs but not with wild-type Tregs (IHC control) (Fig. 5*D* and *SI Appendix*, Fig. S14*A*). In contrast, IHC for effector immune cells revealed that ACT with SRC-3 KO Tregs significantly increased the number of CD4^+^ T cells, CD8^+^ T cells, and NK cells in cancers compared to ACT with wild-type Tregs (Fig. 5 *E*–*G* and *SI Appendix*, Fig. S14 *B*–*D*). Additionally, ACT with SRC-3 KO Tregs markedly reduced Foxp3+ cells in breast tumors compared to ACT with WT Tregs (Fig. 5*H* and *SI Appendix*, Fig. S14*E*). ACT with SRC-3 KO Tregs also significantly increased Ifng and Cxcl9 in breast tumors compared to ACT with wild-type Tregs (Fig. 5 *I* and *J* and *SI Appendix*, Fig. S14 *F* and *G*). Our results obtained with ACT were in accordance with the observations from the SRC-3^d/d^:Treg female mice. Collectively, ACT with SRC-3 KO Tregs results in an increase in the infiltration of immune effector cells and enhances the Ifng/Cxcl9 axis in breast tumors, along with the subsequent generation of an efficient antitumor immune microenvironment that eradicated preexisting E0771 tumors in SRC-3^f/f^ host mice.

Following tumor clearance to undetectable levels by ACT with SRC-3 KO Tregs, recurrence was not detected for at least 215 d (Fig. 5*A*). This observation suggested that ACT with SRC-3 KO Tregs leads to a state of long-term tumor resistance like that seen in SRC-3^d/d^:Treg female mice. To test this hypothesis, E0771:LUC cancer cells were “againreinjected” into tumor-eradicated SRC-3^f/f^ female mice that had received prior ACT with SRC-3 KO Tregs (*SI Appendix*, Fig. S12*C*). The reinjected E0771:LUC cells also did not develop into breast tumors in the tumor-eradicated SRC-3^f/f^ mice after ACT with SRC-3 KO Tregs but did so in control SRC-3^f/f^ mice (Fig. 5*K* and *SI Appendix*, Fig. S13*D*). Therefore, a single ACT injection with SRC-3 KO Tregs supports long-term tumor elimination and resistance in mice.

To define whether current immune checkpoint modulators have a similar tumor eradication effect as SRC-3 KO Tregs, E0771 breast tumor–bearing SRC-3^f/f^ female mice (C57/BL6J-Albino) were treated with an anti-PD-L1 antibody (7.5 mg/kg) or control IgG (7.5 mg/kg) twice a week for 2 wk (*SI Appendix*, Fig. S15*A*) based on a previous study (29). Compared to control IgG treatment, however, anti-PD-L1 antibody treatment did not suppress the growth of E0771 breast tumors in immune-intact mice (*SI Appendix*, Fig. S15 *B* and *C*). Therefore, anti-PD-L1 immunotherapy did not recapitulate the tumor eradication activity of SRC-3 KO Tregs.

## Discussion

Tregs play a critical role in cancer progression by generating an immune-suppressive microenvironment and thus have been a major focus for immune checkpoint inhibitor therapeutic development. While the vast majority of Treg-based immune checkpoint modulators are focused on cell membrane signaling proteins, some efforts to modulate Treg function at a transcriptional level within the nucleus have been pursued. For example, Treg-specific transcriptional coactivator p300 KO mice show an antitumor phenotype due to increased TCR-induced apoptosis of Tregs (30). However, Treg-cell-specific p300 KO mice show incomplete tumor inhibition and develop adverse health effects, such as weight loss, dermatitis, lymphadenopathy, and splenomegaly related to Treg depletion as these animals age (30). In contrast, SRC-3 KO increases Treg proliferation and alters their function by switching them from protumor into antitumor immune cells while not causing systematic autoimmune disease, reproductive dysfunction, shortened lifespan, or lower body weight. These observations demonstrate a benign side effect profile for SRC-3 KO Tregs in mice that contrasts with most immune checkpoint inhibitors and with chimeric antigen receptor (CAR)-T cell therapies in that it avoids adverse effects such as cytokine release syndrome, neurological events, and other potentially serious side effects (31). Notably, our SRC-3-KO cells require only a single injection to achieve a potent antitumor effect. Systemic injection of Treg-cell-targeting antibodies can result in toxicity, including serious autoimmunity in Treg-targeted cancer immunotherapies that involve CTLA-4 blockade (32–34). However, our SRC-3 KO Treg therapy does not cause discernable toxicity. Even though spleen-derived Foxp3^+^SRC-3^−^ Treg cells possess a different gene expression profile than WT Tregs, spleen-derived Foxp3^+^SRC-3^−^ Tregs did not cause any detectable phenotypic changes in SRC-3^d/d^:Treg female mice. Also, our analysis of multiple tumor injections in SRC-3^d/d^:Treg mice showed that SRC-3 KO Tregs retained their antitumor activity for over 175 d in the mice.

SRC-3 KO Tregs express additional proteins including CRE-ERT2 recombinase and GFP proteins. In addition to this, E0771 breast tumors also express the luciferase protein. Although these nonmouse proteins possibly could act as neoantigens and help the body mount an immune response (35, 36), these expressed exogenous genes have been successfully used in numerous cancer studies and appear not to be confounders in immune-related studies.

**Fig. 6.**
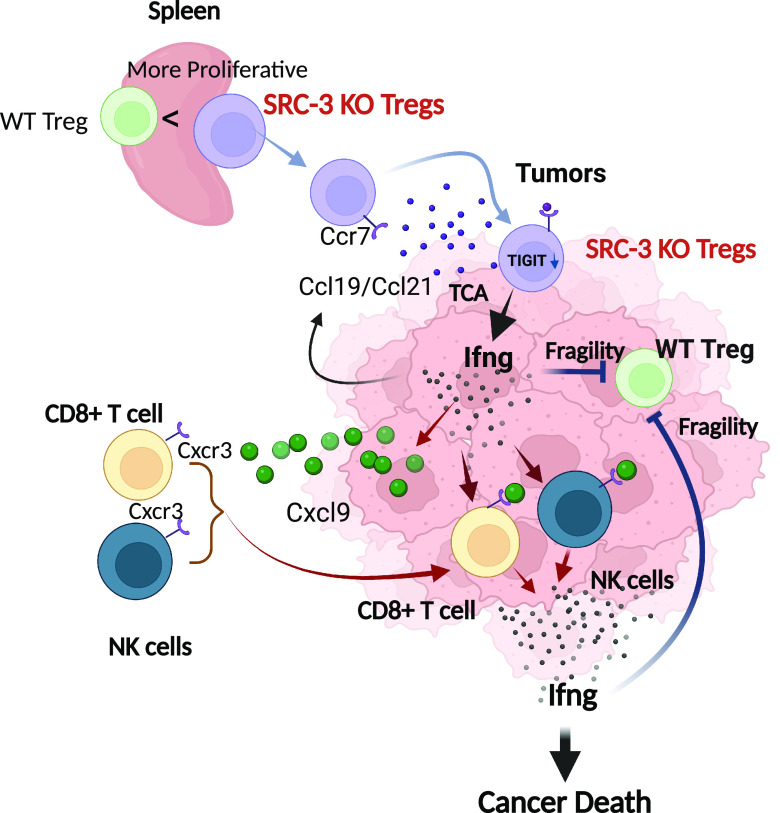
Model for tumor eradication by SRC-3 KO Tregs. SRC-3 KO Tregs are more proliferative than WT in spleens due to increased levels of Tgf-β, Il10, and IL35 in spleens. Compared to WT Tregs, however, SRC-3 KO Tregs have reduced immune suppressive function due to the down-regulation of genes involved in immune suppression, such as TIGIT. Based on the homing activity of Tregs to cancer (Ccl19/Ccl21/Ccr7 axis) (37, 38), SRC-3 KO Tregs more aggressively infiltrate into breast tumors. In breast tumors, SRC-3 KO Tregs induced Ifng expression based on TCR activation. The elevated Ifng causes the fragility of WT Tregs in breast tumors. Also, elevated Ifng induces Cxcl9 expression in breast tumors to actively recruit cytotoxic Cxcr3+ immune cells (such as CD8+ and NK cells) into breast tumors. In addition, the recruited and tumor-resident CD8+ and NK cells activated by elevated Ifng eradicate cancer cells. The graphic was generated by BioRender.

Another surprising but important therapeutic consideration related to the disruption of SRC-3 expression in Tregs is that ACT with SRC-3 KO Tregs eradicated tumors, even though normal Tregs are present in SRC-3^f/f^ recipient mice. This observation raised the question of how SRC-3 KO Tregs take on a functionally dominant role over wild-type Tregs that leads to ACT-mediated tumor eradication. Our hypothesis is shown in Fig. 6. The SRC-3 KO Tregs are more proliferative than WT Tregs in the spleen due to elevated levels of splenic TGF-β, Il-10, and IL35. Compared with WT Tregs, however, SRC-3 KO Tregs do not have heightened immune suppressive functions in the tumor microenvironment due to the down-regulation of many genes involved in immune suppression such as T Cell Immunoreceptor With Ig And ITIM Domains (TIGIT) and others. Furthermore, based on the homing activity of Tregs to cancer, such as from the CCL22/CCR4 axis (37, 38), SRC-3 KO Tregs can aggressively move into breast tumors. After TCR-mediated engagement with tumor antigens, SRC-3 KO Tregs, but not WT Tregs, produce high amounts of Ifng. The elevated levels of Ifng further induce antitumor immune activity in tumors, leading to increased CXCL9 expression from tumor cells; CXCL9 then recruits CXCR3+ immune cells, such as cytotoxic CD8+ T cells and NK cells, into cancers to increase effector immune cell infiltration (39, 40). Elevated Infg activates both recruited and resident CD8+ T cells and NK cells in breast tumors, which can lead to the positive feedback that drives even more Infg production (24, 41). Furthermore, high Ifng production occurs only in the tumor microenvironment and is not elevated in the serum of the animals, avoiding systemic toxicity. Ifng is known to cause wild-type Treg cellular “fragility” (42) along with high Treg production of perforin and granzymes (43). Consistent with this, our CD4 cell proliferation suppression assays indeed reveal that activated SRC-3 KO Tregs suppress WT Treg function. Therefore, the activated Ifng/Cxcl9 axis stimulated by SRC-3 KO Tregs would be expected to suppress WT Tregs in breast tumors by enhancing Ifng-induced cellular fragility in WT Tregs. Also, the up-regulated Ifng/Cxcl9 axis can enhance the T-bet transcription factor in naive CD4+ cells that supports their differentiation into T helper (Th)-1 cells while inhibiting their differentiation into Th-2 and Th17 cells (44, 45). The activated Ifng/Cxcl9 axis generated by SRC-3 KO Tregs also should stimulate the expansion of a proinflammatory Th-1-cell population within the tumor microenvironment to facilitate tumor eradication.

Both the Treg-cell-specific SRC-3 KO mouse and ACT with SRC-3 KO Tregs generated a strong tumor-resistance state. Breast tumor recurrence was never again detected, and subsequent 2nd tumor injections did not induce cancers in Treg-cell-specific SRC-3 KO mice and in mice receiving ACT with SRC-3 KO Tregs after eradication of the first tumor. Our data reveal that SRC-3 KO Tregs are more proliferative and viable than WT Tregs, and CD3/CD28 costimulation significantly increases the viability of SRC-3 KO Treg cells compared to WT Tregs. Thus, SRC-3 KO likely increases the life span of Tregs such that they can maintain tumor resistance in mice long term. SRC-3 KO Tregs also showed increased expression of Il-10, Il-35, and Tgf-β compared to wild-type Tregs obtained from the spleen. TGF-β, IL-10, and IL-35 are known to stimulate the development of an induced regulatory T cell population (46–48). In addition, IL-10 enhances IL-2-stimulated proliferation of both CD4^+^ and CD8^+^ T cells by increasing cell division (49). Also, treatment with IL-35 has been shown to enhance the proliferation of Foxp3^+^CD39^+^ CD4^+^ T cells (50). Therefore, the elevation of Il-10, Il-35, and Tgf-β in SRC-3 KO Tregs is expected to increase/stabilize the number of SRC-3 KO Treg cells in the spleen by autocrine signaling pathways that then support their later infiltration into tumors, leading to tumor eradication.

As discussed in the introduction, SRC-3 is known to broadly function as a primary coactivator for numerous transcription factors in the cell (1). Given its known pleiotropic regulation of gene expression, it is not surprising to us that disruption of the SRC-3 gene results in dramatic changes in the Treg transcriptomic profile that affect the expression of key cytokines and membrane signaling molecules as a group. In addition, studies on the cell-autonomous roles of SRC-3 in cancer cells have revealed that this coactivator also broadly controls multiple transcriptional goal-oriented programs that drive key cancer-promoting pathways underlying cell proliferation, invasion, and metastasis (51–53). Because of these attributes of SRC-3 biology, further studies will need to consider the widespread transcriptional effects of this key coactivator on Treg function. Clearly, this pleiotropic effect cannot be expected to be restricted to the perturbation of only a single or even a few molecular pathways.

In conclusion, we demonstrate that SRC-3 KO Tregs show strong potential as a therapeutic approach for cancer therapy. SRC-3 KO Treg-based ACT therapy also avoids many of the known limitations seen for other immune-therapeutics, such as membrane immune checkpoint inhibitors, and is effective in breast/prostate mouse tumors where checkpoint inhibitor drugs are less effective. Also, it should be pointed out that current CAR-T therapy efficacy for solid tumor treatment has not yet been widely achieved, and significant side effects can limit their use (54). Since SRC-3 KO Tregs do not produce the strong, generalized autoimmune side effects that are typically seen with other immune checkpoint inhibitors, dose-dependent toxicities appear not to limit the dose of SRC-3 KO Tregs in ACT. Finally, SRC-3KO Tregs appear to confer extremely long-lasting protection against cancer recurrence in mice, suggesting that further work to develop genetically altered SRC-3 KO Tregs is warranted as a cell-based therapeutic agent to treat cancer.

## Materials and Methods

### Tamoxifen-Induced Treg SRC-3 Gene KO in Treg Cells.

Tamoxifen (100 mg, Tocris Cat. No. 6432) was dissolved in 5 mL of corn oil (20 mg/mL). Then, 100 µL of the 20 mg/mL tamoxifen solution was injected intraperitoneally into SRC-3^f/f^:Foxp3^Cre-ERT2/+^ bigenic female mice (20 g, 8 wk old) and female control mice (20 g, 8 wk old), daily for 5 d. The final concentration of injected tamoxifen was 75 mg/kg. The tamoxifen treatment of SRC-3^f/f^:Foxp3^Cre-ERT2/+^ mice results in the generation of SRC-3^d/d^:Treg mice.

### Treg Cell Isolation from Spleens and Tumors.

Each mouse spleen was placed over a 70-µm filter on top of 50 mL tubes. Mouse spleens were processed through the filter using a syringe with RPMI1640 supplemented with 10% Fetal Bovine Serum (FBS). The resultant cell suspension was incubated with 1× eBioscience RBC lysis buffer for 5 min on ice and then filtered through a 40-µm mesh to obtain single-cell splenocytes. Splenocytes were counted using a Vi-cell counter. Finally, T cell isolation was performed using Miltenyi Biotec's MACS CD4^+^CD25^+^ T cell isolation kit using the manufacturer's recommended protocol. In this process, positive selection of CD4^+^ CD25^+^ (Tregs) cells was performed using MACS CD25 MicroBeads, and nonselected cells were labeled as CD4^+^ CD25^−^ (Tconv) cells.

Tumor tissue was first minced with scissors and then digested with collagenase IV (3 mg/mL) in RPMI 1640 medium containing 10% FBS at 37 °C for 1 h. Digestion was then further performed by mashing and then filtered through a 40-μm filter using a 10-mL syringe plunger to get a single-cell suspension. After lysis of RBCs, Treg isolation was performed using Miltenyi Biotec's MACS CD4^+^CD25^+^ T cell isolation kit using the manufacturer's recommended protocol.

### RNA Expression Analysis of Genes of Interest in SRC-3 KO Versus WT Treg Cells.

Total mRNA was isolated from Tregs from the spleens and breast tumors of breast tumor–bearing SRC-3^f/f^ and SRC-3^d/d^:Treg female mice using TRI reagent (Sigma-Aldrich T9424) and the GlycoBlue coprecipitation protocol. cDNA was generated from total Treg RNA (~0.5 to 10 µg) using a Vilo SuperScript Master Mix cDNA synthesis kit (Invitrogen catalog number: 11754050). To study relative gene expression, qPCR was carried out using sequence-specific primers and with PowerTrack SYBR green master mix (ThermoFisher, catalog number: A46109). Relative mRNA expression was calculated by the 2^−ΔΔCT^ method of quantitative PCR after normalization with 18S rRNA levels. At least three technical replicates represent each result. Primer sequences are described in *SI Appendix*.

### RT-PCR for Treg Chemotactic Ligands in E0771 Cells upon Ifng Treatment.

E0771 breast cancer cells were cultured with RPMI1640 media containing 10% FBS. When cell confluence was 90%, E0771 breast cells were treated with 0, 1, 10, and 100 ng/mL of active mouse recombinant Ifng protein (Abcam, catalog number: 259378) for 3 d. RPMI1640 media containing Ifng were exchanged every other day. Total mRNA was isolated from E0771 breast cancer cells treated with Ifng using an RNeasy Kit (Qiagen, catalog number: 74104). The cDNA was generated from total Treg RNA (~0.5 to 10 µg) using a Vilo SuperScript Master Mix cDNA synthesis kit (Invitrogen, catalog number: 11754050). To study relative gene expression, qPCR was carried out using sequence-specific primers and PowerTrack SYBR green master mix (ThermoFisher, catalog number: A46109). Relative mRNA expression was calculated by the 2^−ΔΔCT^ method of quantitative PCR after normalization with 18S rRNA levels. At least three technical replicates represent each result. Primer sequences are described in *SI Appendix*.

### Determination of Cxcl9 Expression in E0771 Cells upon Ifng Treatment.

E0771 breast cancer cells were cultured with RPMI1640 media containing 10% FBS. When cell confluence was 90%, E0771 breast cells were treated with 0 and 100 ng/mL of active mouse recombinant Ifng protein (Abcam, catalog number: 259378) for 24, 48, and 72 h. RPMI1640 media containing Ifng were exchanged every other day. As described above, total mRNA and cDNA were generated from E0771 breast cancer cells treated with Ifng. Primer sequences are described in *SI Appendix*.

### Anti-Infg Antibody Treatment of Breast Tumor–Bearing SRC-3^f/f^ and SRC-3^d/d^: Foxp3^Cre/+^ Female Mice.

SRC-3^f/f^ and SRC-3^f/f^:Foxp3^Cre/+^ female mice (8 wk old) were treated with tamoxifen (75 mg/kg, once a day for 5 d). At the end of the 2nd week after the final tamoxifen treatment, SRC-3^f/f^ and SRC-3^d/d^: Treg female mice were intraperitoneally treated with an anti-mouse Ifng antibody (BioXCell, catalog number: DE0055, 0.2 mg/kg, twice a week for 2 wk) or rat IgG (BioXCell, catalog number: BE00884, 0.2 mg/kg, twice a week for 2 wk) as controls. On the third day after the first antibody treatment, E0771 cells (1 × 10^5^ cells) were orthotopically injected into the mammary glands of SRC-3^f/f^ and SRC-3^d/d^:Treg female mice. Tumor luciferase activity was determined with an IVIS bioluminescent imager.

### Treg Proliferation Assay.

Treg cells were purified from splenocytes collected from the SRC-3^f/f^:Foxp3^Cre-ERT2/+^ bigenic and SRC-3^f/f^ mice 2 wk after the last injection of tamoxifen, using a Miltenyi Biotec’s MACS CD4+CD25+ T cell isolation kit according to the manufacturer’s protocol. Tregs were seeded to anti-CD3 antibody precoated 48-well plates at a density of 3 × 10^5^cells/mL in the stimulation medium (RPMI+10% FBS+1% anti-anti and supplemented with 2 µg/mL anti-CD28 antibody and 1,000 U/mL mouse IL-2) for 2 d. On day 3, half of the medium was replaced with fresh maintenance medium (RPMI+10% FBS+1% anti-CD28 and supplemented with 1,000 U/ml mouse IL-2). Cell viability and density were detected by trypan blue staining on days 0, 3, 5, and 7 with the Bio-Rad TC20 cell counter.

### CD4 Cell Proliferation Suppression Assays (Mixed Treg and Tconv Experiments).

Tregs and Tconv cells were isolated as described in the “Mouse splenocytes and Treg cell isolation” section. CD4+CD25-(Tconv) cells were washed with 1× PBS and then incubated with Cell Trace Violet (CTV, ThermoFisher, catalog # C34557) solution in 1× PBS at a concentration of 5 mM per 1 × 10^6^ cells for 30 min at 37 °C for staining. Serum-enriched media were added to quench any unreacted CTV and then centrifuged at 300 rpm for 5 min at 40 °C. Stained Tconv cells were resuspended in enriched activation media (RPMI1640 + 10% FBS + 50 µM mercapto ethanol + 2 µM CD28 (0.5 mg/mL) + 200 U IL2 (100 µg/mL) at a concentration of 5 × 10^4^ cells/mL. Unstained Tregs were resuspended in enriched activation media at a concentration of 1 × 10^6^ cells/mL. Tregs were mixed with stained Tconv cells at different ratios and then plated in a CD3 (5 µg/mL) precoated U-bottom 96-well bottom plate with incubation at 37 °C. The proliferation of stained Tconv cells was measured using flow cytometry on days 3, 4, and 5.

### Quantification and Statistical Analysis.

Data are presented as the mean ± SEM values. Statistical comparisons between two groups were performed with one-way ANOVA, followed by the Student *t* test or the Mann–Whitney *U* test. Sidak's multiple comparison test was used to compare more than two groups. A *P* value < 0.05 was considered significant. Prism 8.0 software was used for statistical analyses. The differential RNA expression profile between SRC-3 KO Treg versus wild-type Treg was analyzed using R, Bioconductor, and orange program. The cellular pathways associated with SRC-3 KO Treg cells were analyzed with DAVID and GSEA programs.

## Supplementary Material

Appendix 01 (PDF)

## Data Availability

The accession numbers for the RNA-seq data from SRC-3 KO and wild-type Tregs in this paper are GEO: GSE216931 (18). All study data are included in the article and/or *SI Appendix*.
