# Supplementary material for: Steroid receptor coactivator 3 is a key modulator of regulatory T cell–mediated tumor evasion
Source: Proc Natl Acad Sci U S A. 2023 May 30;120(23):e2221707120. doi: 10.1073/pnas.2221707120 (PMC10266015; doi:10.1073/pnas.2221707120)
Supplement: Supplementary file 1 — Appendix 01 (PDF) [file pnas.2221707120.sapp.pdf]

Supporting Information for

Steroid Receptor Coactivator-3 is a Key Modulator of Regulatory Cell-Mediated Tumor Evasion

Sang Jun Han[1,2,*], Prashi Jain[1], Yosef Gilad[1], Yan Xia[1], Nuri Sung[1], Mi Jin Park[1], Adam M. Dean[1], Rainer B. Lanz[1], Jianming Xu[1,2], Clifford C. Dacso[1,2,3], David M. Lonard[1,2], and Bert W. O'Malley[1,2,*]

[1]Department of Molecular Cellular Biology, Baylor College of Medicine, TX, 77030, USA.

[2]Dan L. Duncan Cancer Center, Baylor College of Medicine, TX, 77030, USA

[3]Department of Medicine, Baylor College of Medicine, TX, 77030, USA

*Corresponding Authors:

Sang Jun Han

Department of Molecular Cellular Biology, Baylor College of Medicine, TX, 77030, USA.

E-mail: sjhan@bcm.edu

Phone: 713-798-6276

Bert W. O'Malley

Department of Molecular Cellular Biology, Baylor College of Medicine, TX, 77030, USA.

E-mail: berto@bcm.edu

Phone: 713-798-6205

This PDF file includes:

Materials and Methods

Figures S1 to S15

Tables S1 to S2

Abbreviation

SI References

Materials and Methods

***Animal Studies*:** C57BL/6J (RRID:IMSR_JAX:000664) and Foxp3 $^{tm9(EGFP/Cre/ERT2)Ayr}$ mice (RRID:MGI:5288172), and Foxp3$^{tm4(YFP/icre)Ayr}$/J (RRID:IMSR_JAX:016959) were purchased from Jackson Laboratory. Floxed SRC-3 (SRC-3$^{f/f}$) mice employed to knock out the SRC-3 gene in specific tissues were described before (1). All animal studies were conducted with the approval of the Institutional Animal Care and Use Committee at Baylor College of Medicine. All mice were euthanized in a CO_2 chamber. Additionally, any animals that showed a loss of mobility and weight loss were euthanized. Cancers were not allowed to grow to >20% of body weight or ulcerate. At the end of experiments, all animals were euthanized, and cancers were harvested at a volume of no more than 4,000 mm^3.

Orthotopic injection of E0771:LUC cells into the mammary fat pads of syngeneic female recipient mice. A total of 1 × 10^5 of E0771:LUC cells were resuspended in 50 µL of phosphate-buffered saline (PBS) and then injected into one of the 4th mammary fat pads in SRC-3$^{f/f}$ (eight weeks old, n=8) and SRC-3$^{d/d}$:Treg (eight weeks old, n=8), as described in our previous studies (2). Tumor growth was monitored by evaluating luciferase activity using an *In Vivo* Imaging System (IVIS, Xenogen). Luciferase activity in E0771 tumors was assessed twice a week using IVIS.

IVIS analysis of luciferase bioluminescence in mice. Mice were anesthetized with a 1.5% isoflurane/air mixture using an inhalation anesthesia system (VetEquip). Next, D-Luciferin (Xenogen, Cat# 122799) was injected intraperitoneally at 40 mg/kg mouse body weight. Ten minutes after D-luciferin injection, mice were imaged using IVIS under continuous exposure of

1% to 2% isoflurane. Imaging parameters were maintained for comparative analysis. Grayscale and pseudocolor images showing bioluminescence were superimposed and analyzed using Living Image software (Version 4.4, Xenogen). A region of interest (ROI) was manually selected over the relevant areas showing a luciferase signal. The ROI area was kept constant across the experiments, and the luciferase activity was recorded as total luciferase photon counts per second per cm^2 within the ROI.

Injection of a second round of E0771:LUC cells into tumor-eradicated SRC-3$^{d/d}$:Treg female mice. Seven weeks after the first injection of E0771:LUC, tumor-eradicated SRC-3$^{d/d}$:Treg female mice (Figure 2A, n=5) were orthotopically injected with E0771:LUC cells (1 x10^5) in their mammary fat pads. As controls, SRC-3$^{f/f}$ female mice (eight weeks old, n=5) were orthotopically injected with E0771:LUC cells (1 x10^5) into their mammary fat pads. Luciferase activity of tumors was determined twice a week by IVIS imaging.

ACT of Treg cells. The SRC-3$^{f/f}$:Foxp3$^{Cre\text{-}ERT2/+}$ bigenic female mice (eight weeks old, n=9) and SRC-3$^{f/f}$ female mice (eight weeks old, n=9) were treated with tamoxifen (75 mg/kg) daily for five days. After two weeks, SRC-3 KO Tregs were isolated from the spleens of SRC-3$^{d/d}$:Treg mice. As controls, wild-type Tregs were isolated from the spleens of SRC-3$^{f/f}$ mice treated with tamoxifen. The SRC-3$^{f/f}$ mice (eight weeks old, n=18) were orthotopically injected with E0771:LUC cells (1 x10^5) into mammary fat pads. Seven days after injection of E0771:LUC cells, isolated SRC-3 KO Tregs (cell number: 200 ~ 800 K cells) in 50 μL of MDEM/F12 media were given to tumor-bearing littermate SRC-3$^{f/f}$ mice by retro-orbital injection (n=9). As controls, wild-type Tregs (cell number: ~900 K cells, cell viability: ~90%) in 50 μL of

MDEM/F12 media were given to tumor-bearing littermate SRC-$3^{f/f}$ female mice by retro-orbital injection (n=9). After ACT, tumor luciferase activity was determined in mice adoptively transferred with SRC-3 KO versus wild-type Tregs.

Second round of E0771 cell injections into SRC-3 KO ACT Treg recipient mice. Forty days after the first round of E0771 cell injections, tumor-eradicated SRC-$3^{f/f}$ mice that received ACT of SRC-3 KO Tregs (n=3) were orthotopically injected with E0771:LUC cells ($1x10^5$) into a mammary fat pad. As controls, SRC-$3^{f/f}$ female mice (eight weeks old, n=2) were orthotopically injected with E0771 cells ($1x10^5$) into a mammary fat pad. Tumor luciferase activity was determined twice a week by IVIS imaging.

Mouse cytokine/chemokine analysis with tumors. Breast tumors were isolated from SRC-$3^{f/f}$ (n=4) and SRC-$3^{d/d}$:Treg female mice (n=4) on day 14 after E0771 cell injection. Cytokine and chemokine levels in breast cancers were determined using Proteomic Profile Mouse Cytokine Array Kit (R&D System, ARY0066). Afterward, cytokine levels were quantified with the ImageJ program (3).

Generation of Treg cell-specific SRC-3 KO mice. SRC-$3^{f/f}$ mice originate from a *mixed* C57BL/6 x *129* genetic background (1). SRC-$3^{f/f}$ female mice were crossed with Foxp$3^{tm4(YFP/icre)Ayr}$/J male mice to generate Treg cell-specific SRC-3 KO bigenic mice (SRC-$3^{d/d}$:Foxp$3^{tm4(YFP/icre)Ayr/+}$). The genotyping of the SRC-$3^{f/f}$ mice was conducted with primers (GAAACCTCAAGGTTATCCTTCAATT and TGCTCTGCTTAGATACCTGCTG) as previously reported (1). Genotyping of Foxp$3^{tm4(YFP/icre)Ayr/+}$ mice was conducted using a protocol

published by the Jackson Laboratory (https://www.jax.org/Protocol?stockNumber=016959&protocolID=36057). To generate the syngeneic mouse model of breast cancer with E0771 breast cells that spontaneously generated from C57BL/6J female mice, SRC-$3^{f/f}$ female mice (mixed background) were mated with C57BL/6J male mice, and genotyping of the SRC-$3^{f/f}$ mice were conducted with primers (GAAACCTCAAGGTTATCCTTCAATT and TGCTCTGCTTAGATACCTGCTG) as previously reported (1). After nine generations of mating to C57BL/6J males, SRC-$3^{f/f}$ mice were established on a pure C57BL/6J background. Further, Jackson Laboratory confirmed that the genetic background of the $Foxp3^{tm9(EGFP/Cre/ERT2)Ayr}$ ($Foxp3^{Cre\text{-}ERT2/+}$) strain is in a pure C57BL/6J background. To further ensure a uniform genetic background, $Foxp3^{Cre\text{-}ERT2/+}$ mice were also mated with C57BL/6J mice for nine generations to generate a$Foxp3^{Cre\text{-}ERT2/+}$ mice in a pure C57BL/6J background. Genotyping of $Foxp3^{Cre\text{-}ERT2/+}$ mice was conducted using a protocol published by the Jackson Laboratory (https://www.jax.org/Protocol?stockNumber=016961&protocolID=28173). SRC-$3^{f/f}$ female mice were mated with $Foxp3^{Cre\text{-}ERT2/+}$ male mice to produce SRC-$3^{f/f}$:$Foxp3^{Cre\text{-}ERT2/+}$ bigenic mice.

Determination of female reproductive toxicity resulting from Treg cell-specific SRC-3 KO. SRC-$3^{f/f}$:$Foxp3^{Cre\text{-}ERT2/+}$female mice (eight weeks old, n=5) and SRC-$3^{f/f}$ female mice (eight weeks old, n=5) were treated with tamoxifen (75 mg/kg) daily for five days. Three weeks after tamoxifen treatment, each mouse in both groups was mated with a fertility-proven male mouse (C57BL/6J, eight weeks old) at a 1:1 ratio. The pregnancy rate and pup numbers per female mouse were then determined.

Animal numbers and power calculations**.** A tumor luciferase activity assay indicated that tumor luciferase activity was not detected in SRC-3$^{d/d}$:Treg female mice compared to control mice (Figure 2A). Therefore, a power calculation based on these data revealed that the animal size (n=1/group) was sufficient to see a significant effect on tumor eradication by SRC-3 KO Treg cells in mice ($p<0.001$). We chose to use four or more mice in each group for all mouse experiments to achieve additional statistical power.

Culture of the E0771 and RM-1cell lines**.** E0771 mouse mammary gland carcinoma cells (ATCC Cat# CRL-3461, RRID: CVCL_GR23) and RM-1 mouse prostate cancer cells (ATCC Cat# CRL-3310, RRID: CVCL_B459) cells were grown in RPMI 1640 containing 10% (vol/vol) fetal bovine serum (FBS) in a humidified incubator with 5% (vol/vol) CO_2 at 37 °C.

Generation of luciferase-labeled E0771 and RM-1 cells**.** The firefly luciferase cDNA was cloned into the PSMPUW-Hygro lentiviral vector (Cell Biolabs, catalog number: VPK-214). Lentiviruses containing the luciferase expression cassette were produced in 293 TN cells (System Bioscience, catalog number: LV900A-1) by transient transfection with the PSMPUW-Hygro lentiviral vector carrying the luciferase gene and the ViraSafe™ Lentiviral Packaging System (Cell Biolabs, catalog number: VPK-206) with Lipofectamine 2000 (Thermo Fisher Scientific, catalog number: 11668030). Recombinant lentivirus titer was measured using Lenti-XTM GoStixTM Plus (ClonTech, catalog number: 631280). E0771 and RM-1 cells were transduced with lentiviral vectors carrying the luciferase expression cassette with TransDux MAX^{TM} (System Bioscience, catalog number: LV860A-1). Luciferase-labeled E0771 and RM-1

cells were then selected in the presence of 300 µg/mL hygromycin. Luciferase gene expression in E0771 and RM-1 cells was validated using a luciferase activity assay kit (Promega). Luciferase-labeled E0771 and RM-1 cells were maintained in RPMI 1640 media supplemented with 10% FBS, penicillin/streptomycin, and 300 µg/mL hygromycin.

Orthotopic injection of luciferase-labeled prostate cancer cells into the prostates of syngeneic male recipient mice. A total of 1×10^3 luciferase-labeled RM-1(RM-1:LUC) cells were resuspended in 50 µL of phosphate-buffered saline (PBS) and then orthotopically injected into the ventral lobe of the prostate in SRC-$3^{f/f}$ (n=3, eight weeks old) and SRC-$3^{d/d}$:Treg male mice (n=3, eight weeks old), as described in previous studies (4). Tumor growth was monitored by evaluating luciferase activity using an IVIS imaging system. Luciferase activity in RM-1 prostate cancers was assessed twice a week.

Prevention of tumor initiation in SRC-$3^{d/d}$:Treg female mice. SRC-$3^{f/f}$:Foxp$3^{Cre\text{-}ERT2/+}$ (eight weeks old,n=2) and SRC-$3^{f/f}$ female mice (eight weeks old, n=2) were treated with tamoxifen (75 mg/kg) daily for five days. After two weeks, E0771:LUC ($1x10^5$) cells were orthotopically injected into the mammary fad fat of one SRC-$3^{d/d}$:Treg female mouse. As controls, E0771:LUC ($1x10^5$ cells) were orthotopically injected into one SRC-$3^{f/f}$ female mice. Tumor luciferase activity was determined twice a week by IVIS imaging.

Repression of pre-existing E0771 tumors in mice. SRC-$3^{f/f}$:Foxp$3^{Cre\text{-}ERT2/+}$ (eight weeks old, n=3) and SRC-$3^{f/f}$ female mice (eight weeks old, n=4) were orthotopically injected with $1x10^5$ E0771:LUC cells per mouse. On day seven, after cell injections, tumor-bearing SRC-$3^{f/f}$:Foxp$3^{Cre\text{-}ERT2/+}$ and SRC-$3^{f/f}$ female mice were treated with tamoxifen (75 mg/kg) daily for

five days. Afterward, the tumor luciferase activity was determined twice a week by IVIS imaging.

Tamoxifen and vehicle treatment of E0771 breast tumor-bearing SRC-3$^{f/f}$ female mice. E0771 breast cancer cells ($1x10^5$ cells) were orthotopically injected into SRC-3$^{f/f}$ female mice (8 weeks old). On the 9th day after breast cancer cell injection, tumor-bearing mice were treated with tamoxifen (75 mg/kg, once a day for 5 days) or vehicle as controls. Tumor luciferase activity was determined with an IVIS bioluminescent imager and quantified with Living Image software (version 4.7.4).

Dual Immunofluorescence Staining. Tissue slices were deparaffinized by sequential soaking in xylene, ethanol and water. Antigen retrieval was performed with antigen unmasking solution (Vector Laboratory, catalog number: H-3300). Slides were blocked with 2.5% (w/v) normal goat serum to reduce nonspecific antibody binding. Slices were incubated with anti-SRC-3 (Cell Signaling Technology Cat# 2126, RRID:AB_823642, 1:100 dilution) and anti-FOXP3 (rat, LSBio, catalog number: LS-C344878, 1:100 dilution) antibodies at 4 °C overnight. Afterward, slides were washed with TBST [20 mM Tris (pH 7.5), 150 mM NaCl and 0.1% (w/v) Tween 20] and then incubated with goat anti-rabbit Alexa Fluor 488 (Thermo Fisher Scientific Cat# A-11008, RRID:AB_143165, 1:500 dilution) and goat anti-rat Alexa Fluor 594 (Thermo Fisher Scientific Cat# A-11007, RRID:AB_10561522, 1:500 dilution) for 1 h at room temperature. Afterward, slices were washed with TBST and mounted with Antifade Mounting Media containing DAPI (Vector, catalog number: H-2000).

Mouse splenocytes isolation and immunophenotyping. Single mouse spleens were suspended in 2.6 mL of RPMI1640 media (10% FBS, 1% anti/anti) in C-tubes. Each tube was treated with 375 µg of DNAase I (ThermoFisher, Cat# EN0521) and 1.87mg of Collagenase IV (Sigma-Aldrich, Cat# C4-28) in 300 µL of total media and placed on GentleMACS dissociator (Miltenyi Biotec, Cat# 130-095-235) for 15 mins (2x after 10 mins interval). Then, 400 µL of stopping buffer (1X PBS, 0.1M EDTA) was added and centrifuged for a minute at 100 rpm to collect the liquid at the bottom. The resulting single cell suspension was filtered (40 µm mesh), RBC lysed using 200 µL of 1X Ebioscience Lysis solution (Thermo fisher catalog number: 00-4300-54) for five minutes and centrifuged for six minutes at 600x g. The supernatant was discarded, and the cell pellet was suspended in 100 µL of complete media, and 50 µL of single cell suspension was used for cell counting using Vi-cell. Each sample was Fc blocked before staining using 50 µL of 2X FC Block Solution (FACS Buffer, 1:125 dilution of anti-CD16/CD32 (BD) and incubated for 10 minutes on ice, 100 µL of cold FACS buffer was added and centrifuged for six mins at 500x g. The pellet was suspended in FACS buffer and stained using appropriate antibodies for panel 1 (lymphoid panel) and panel 2 (myeloid panel). Single-cell splenocyte suspensions were analyzed using a BD LSRII for the antibody panel by the Cell Cytometry and Sorting Core at Baylor College of Medicine in collaboration with the International Mouse Phenotyping Consortium. Surface antibodies included in this study are—CD5, CD4, CD44, CD8, CD25, CD161, CD62L, CD19, Ly6C, Ly6G, CD21, CD35, CD11b, CD11c, IA-E and CD23.

RNAseq analysis of SRC-3 KO Tregs. Tregs were isolated from spleens from SRC-3$^{f/f}$ female mice and Treg cell-specific SRC-3 KO bigenic mice (SRC-3$^{d/d}$:Foxp3$^{tm4(YFP/icre)Ayr/+}$). RNA was isolated from Treg cells using a Qiagen RNAasy micro-RNA isolation kit. The isolated RNA

was tested for quality on an Agilent 2100 Bioanalyzer and quantitated using a NanoDrop Spectrophotometer. Low-input RNA-seq was conducted using a Takara SMART-seq v4 ultra-low-input RNA kit using the manufacturer's recommended protocol. In brief, purified RNA was incubated with lysis buffer for five minutes, 3-SMART-seq CDS primer II and V4 oligonucleotides were added for first stranded cDNA synthesis. cDNA was amplified using PCR Primer II A and subsequently purified using Ampure XP beads (Beckman). Illumina libraries were prepared using Nextera XT DNA library preparation kits (Illumina) and sequenced using an Illumina Novaseq 6000. The RNA-seq data is pair-end sequenced with 150 bp read depth. The raw FASTAseq files were imported to the NGS biodata base. The cellular pathways enriched in SRC-3 KO Treg cells compared to WT Treg cells were analyzed with the Reactome pathway database.

Anti-PD-L1 antibody treatment of E0771 breast tumor-bearing SRC-3$^{f/f}$ female mice. E0771 breast cancer cells (1x10^{5} cells) were orthotopically injected into SRC-3$^{f/f}$ female mice (8 weeks old). On the ninth day after breast cancer cell injection, tumor-bearing mice were treated with anti-PD-L1 antibody (100 μg/20g mouse, every third day totaling 4 treatments) and control rat IgG (100 μg/20g mouse, every third day totaling 4 treatments) based on a previous study (5). Tumor luciferase activity was determined with an IVIS bioluminescent imager. The luciferase activity was quantified with Living Image (version 4.7.4) software.

IHC analysis. Fresh mouse tumor tissues were fixed with 10% buffered formalin and then embedded in paraffin for subsequent sectioning. Tissue slices were deparaffinized by sequential soaking in xylene, ethanol, and water. Antigen retrieval was performed with antigen unmasking

solution (Vector Laboratory, catalog number: H-3300). Endogenous peroxidase activity was blocked with a 3% (vol/vol) hydrogen peroxide solution. Sections were blocked with 2.5% (w/v) normal goat serum to reduce nonspecific antibody binding. Slices were incubated with primary antibodies, including anti-CD4 (Abcam Cat# ab183685, RRID:AB_2686917, 1:300 dilution), and anti-CD8 (Abcam Cat# ab217344, RRID:AB_2890649, 1:300 dilution), anti-CD49b (Abcam Cat# ab181548, RRID:AB_2847852, 1:300 dilution), IFNG (rabbit, Novus, catalog number: NBP2-66900, 1:100 dilution), CXCL9 (Thermo Fisher Scientific Cat# PA5-81371, RRID:AB_2788585, 1:100 dilution), EGFP (Bioss Cat# bs-2194R, RRID:AB_10881247, 1:100 dilution), and anti-Foxp3 (Thermo Fisher Scientific Cat# PA1-16876, RRID:AB_568540, 1:300 dilution) antibodies at 4 °C overnight. Slices were then stained with HRP polymer-conjugated secondary antibodies (Vector Laboratories, catalog number: 7401 for rabbit antibodies). Slices were reacted with freshly prepared DAB solution (Dako, catalog number: K3468). After staining, nuclear counterstaining was performed with Mayer's hematoxylin. Quantification of IHC signal was conducted with QuPath software (6).

Flow Cytometry Analysis for SRC-3 KO Tregs. Spleens were dissected from mice and rinsed with cold PBS. Spleens were directly mashed through a 100 μm filter using the plunger from a 10 ml syringe. After centrifuge, the pellet was incubated with RBC lysis buffer for 5 min on ice to remove red blood cells (RBC) and then used for Treg isolation with a CD4+CD25+ Treg isolation kit (Miltenyi Biotec, cat # 130-091-041), according to the manufacturer’s instructions. Isolated Tregs were fixed and permeabilized using the Foxp3 Transcription Factor Fixation/Permeabilization buffer (eBioscience) and then incubated with anti-FOXP3-APC (ThermoFisher, catalog number: 17-5773-80) antibody and anti-CD25-FITC (BioLegend,

catalog number: 102005) at 4°C overnight. Cells were washed twice with PBS before flow analysis. Flow cytometry acquisition was performed on an LSRII cytometer (BD), and the collected data was analyzed using FlowJo v10.8.1 software (TreeStar).

Primer sequence.

Primer sequences are as follows for each cytokine: SRC-3 exon 11 (GTCCCAACCAGCAGAACATC, GAAGCAAAGGAAAACGCAGC), Il-2ra (GGATGGGAATCACAAAGCTC, CCAGGGATCAGAAGGAAACA), Ifng (CGGCACAGTCATTGAAAGCCTA, GTTGCTGATGGCCTGATTGTC), Il-10 (AAGGCAGTGGAGCAGGTGAA, CCAGCAGACTCAATACACAC), Il-35 (GCTCCCCTGGTTACACTGAA, ACGGGATACCGAGAAGCAT), Tgfβ (TGATACGCCTGAGTGGCTGTCT, CACAAGAGCAGTGAGCGCTGAA), Tigit (GAATGGAACCTGAGGAGTCTCT, AGCAATGAAGCTCTCTAGGCT), Klrbic (ATGGACACAGCAAGTATCTACCT, AGCTCTCAGGAGTCACTTTATCT), Klrk1 (ACTCAGAGATGAGCAAATGCCATAA, CAGGTTGACTGGTAGTTAGTGCTAAT), Ccr7 (GACCCAGGTGTGCTTCTG, GGCCCAGAAGGGAAGAATTAG),
Ccl2 (GCTCAGCCAGATGCAGTTA, TCACACTGGTCACTCCTACA), Ccl20 (TTGCTTTGGCATGGGTACT, ACTCTTAGGCTGAGGAGGTT), Ccl19 (GGTGCTAATGATGCGGAAGA, GCTGTTGCCTTTGTTCTTGG), Ccl21 (TGCAAGAGAACTGAACAGACAC, TTCCCTGGGAGACACTCTTT), Ccl11 (ACCTTGTGCAGGCAGTTT, GGATGGAGCCTGGGTGA), Cxcl9 (ACTCCAACACAGTGACTCAATAG, CGTTCTTCAGTGTAGCAATGATTT), and 18S rRNA (TCCGATAACGAACGAGACTC, CAGGGACTTAATCAACGCAA).

Supplement Figures and Figure legends

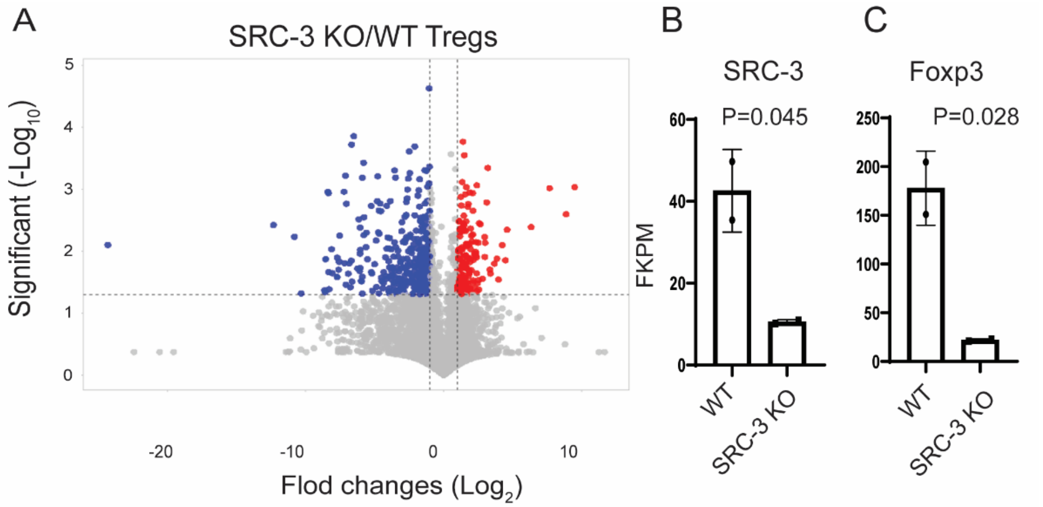

D Downregulated Interleukin signaling in SRC-3 KO Tregs

Pathway identifier	Pathway name	Entities pValue	Entities FDR	Submitted entities found
R-HSA-380108	Chemokine receptors bind chemokines	0.0013	0.4817	Cx3cl1;Ccl5;Cxcr3;Ccr9;Xcl1
R-HSA-449147	Signaling by Interleukins	0.0194	0.5765	Lyn;Il12rb1;Il13ra1;Cx3cl1;Cd80;Il17re;Rorc;Il18rap;Fscn1;Rora;Irs2;Fos;Cebpd;Elk1;Smad3;Il18r1;Ccl5;Anxa2;Sos1;Il9r
R-HSA-6785807	Interleukin-4 and Interleukin-13 signaling	0.0372	0.5765	Il13ra1;Rorc;Fscn1;Rora;Fos;Cebpd
R-HSA-9012546	Interleukin-18 signaling	0.0385	0.5765	Il18r1;Il18rap
R-HSA-2173788	Downregulation of TGF-beta receptor signaling	0.0447	0.5765	Smad3;Nedd4;Tgfbr1

E Upregulated Interferon signaling in SRC-3 KO Tregs

Pathway identifier	Pathway name	Entities pValue	Entities FDR	Submitted entities found
R-HSA-909733	Interferon alpha/beta signaling	0.0027	0.3841	Stat1;Usp18;Isg15;Xaf1
R-HSA-912694	Regulation of IFNA/IFNB signaling	0.0053	0.4133	Stat1;Usp18
R-HSA-1059683	Interleukin-6 signaling	0.0192	0.4133	Stat1
R-HSA-1169408	ISG15 antiviral mechanism	0.0204	0.4133	Stat1;Usp18;Isg15
R-HSA-1169410	Antiviral mechanism by IFN-stimulated genes	0.0303	0.4133	Stat1;Usp18;Isg15

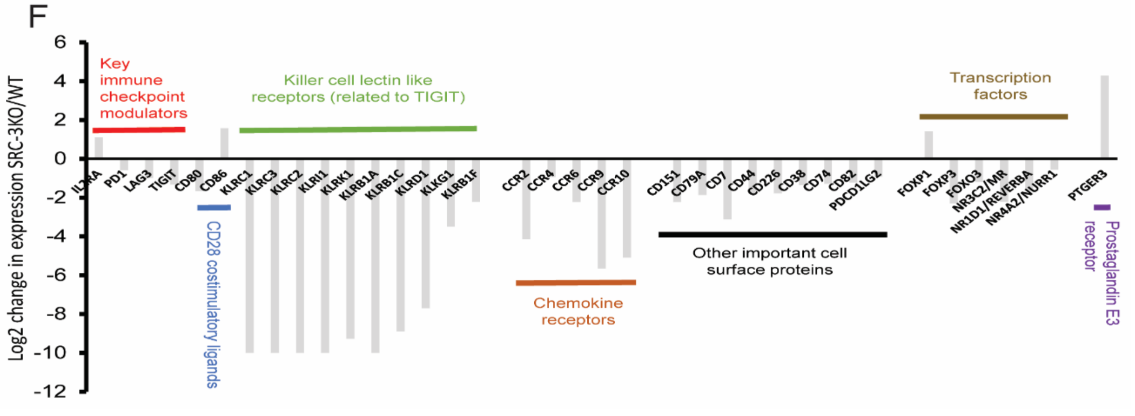

Fig. S1 Downregulation of immune genes in SRC-3 KO Tregs. (A) Volcano plot analysis of differential RNA expression between SRC-3 KO Tregs from SRC-3$^{d/d}$:Foxp3$^{tm4(YFP/icre)Ayr/+}$ female mice and WT Tregs from SRC-3$^{f/f}$ female mice determined by RNA-seq analysis. Genes colored in red were elevated in SRC-3 KO Tregs (> 2-fold, $P<0.05$), and genes colored with blue were significantly down-regulated in SRC-3 KO Tregs (> 2-fold, $P<0.05$) compared to WT Tregs. (B and C) SRC-3 (B) and Foxp3 (C) levels in between WT versus SRC-3 KO Tregs. (D) Downregulated interleukin signaling in SRC-3 KO Tregs. (E) Upregulated Interferon signaling in SRC-3 KO Tregs. (F) Dysregulated gene expression profile of SRC-3 KO Tregs compared to WT Tregs. The relative fold change of each target gene was calculated from the ratio of SRC-3 KO Treg to WT Treg mRNA expression.

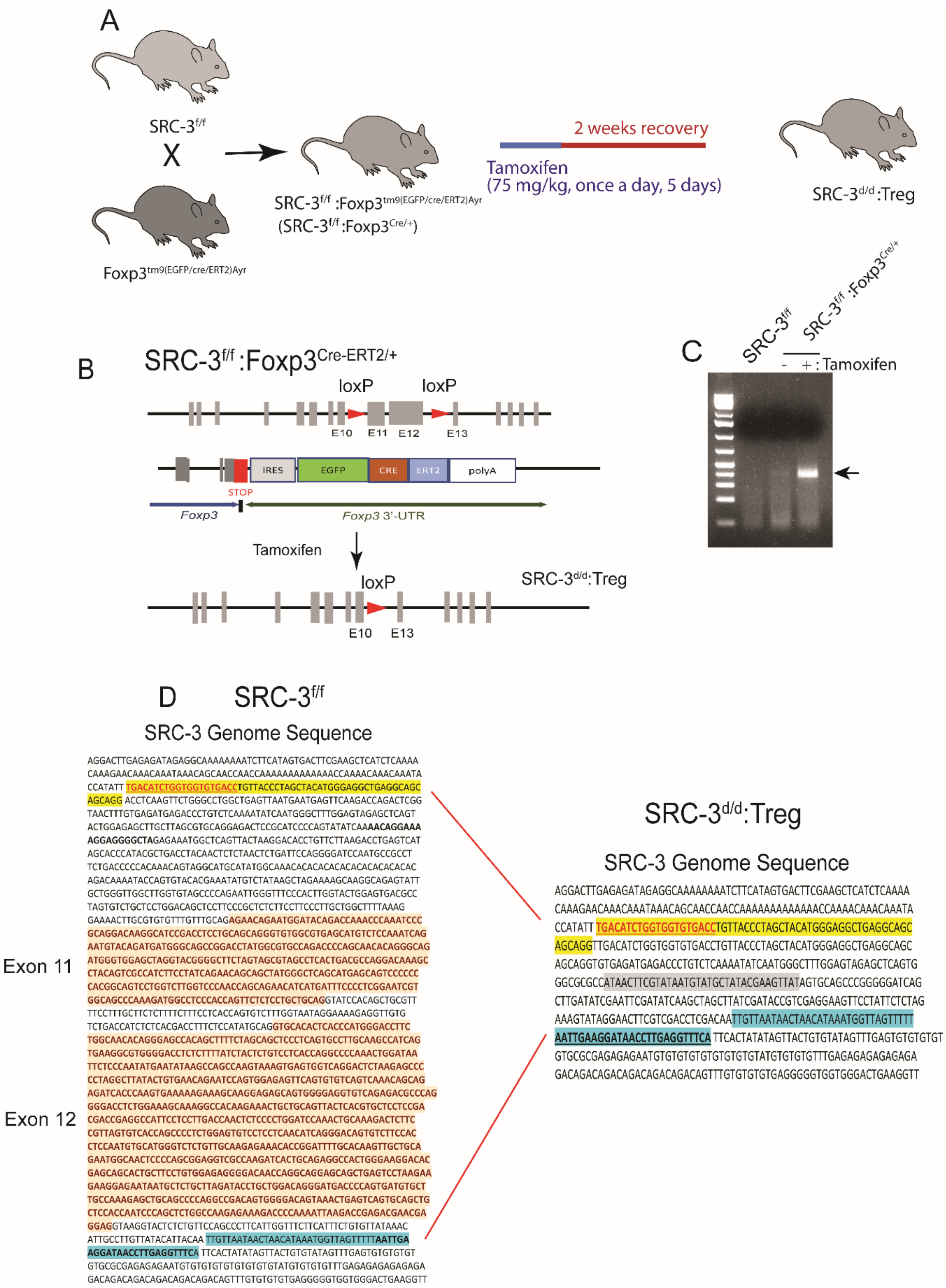
A
SRC-3f/f
X
Foxp3tm9(EGFP/cre/ERT2)Ayr
SRC-3f/f :Foxp3tm9(EGFP/cre/ERT2)Ayr
(SRC-3f/f:Foxp3Cre/+)
2 weeks recovery
Tamoxifen
(75 mg/kg, once a day, 5 days)
SRC-3d/d:Treg
B
SRC-3f/f :Foxp3Cre-ERT2/+
loxP
loxP
E10
E11
E12
E13
IRES
EGFP
CRE
ERT2
polyA
STOP
Foxp3
Foxp3 3'-UTR
Tamoxifen
loxP
SRC-3d/d:Treg
E10
E13
C
SRC-3f/f
SRC-3f/f :Foxp3Cre/+
- + : Tamoxifen
D
SRC-3f/f
SRC-3 Genome Sequence
Exon 11
Exon 12
SRC-3d/d:Treg
SRC-3 Genome Sequence

Fig. S2 Generation of Treg cell-specific SRC-3 KO mice.

(A) Schematic diagram of the generation of Treg cell-specific SRC-3 KO mice (SRC-$3^{d/d}$:Treg). (B) Diagram of SRC-3 gene deletion in Tregs. Cre-ERT2 deletes exons 11 and 12 in the SRC-$3^{f/f}$ mouse specifically in Foxp3 expressing cells upon tamoxifen treatment under the control of the $Foxp3^{tm9(EGFP/Cre/ERT2)Ayr}$ allele in bigenic mice. (C) Genotyping PCR with genomic DNA from Tregs obtained from the spleen of SRC-$3^{f/f}$ and SRC-$3^{f/f}$:$Foxp3^{Cre\text{-}ERT2/+}$ mice treated with either tamoxifen or vehicle and with primers mapping to exon 11 of the SRC-3 gene. (D) DNA sequence analysis of exon 11 and exon 12 showing deletion of these exons in the SRC-3 gene. The PCR product in Panel (C) was isolated and sequenced. Exon 11 and exon 12 (orange highlight) were deleted, and one loxP site (highlighted in gray) exists in the PCR product.

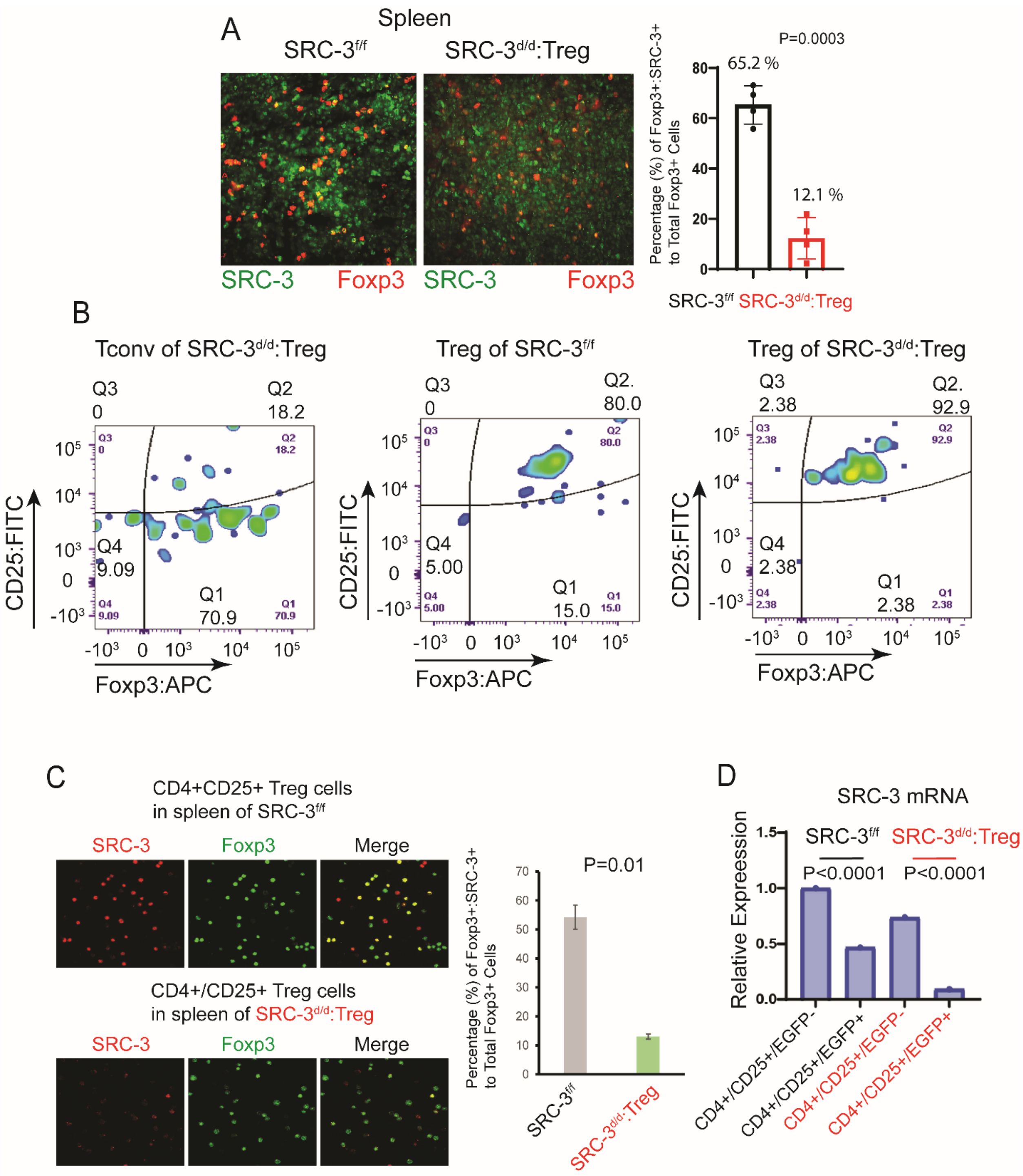

Fig. S3 Validation of SRC-3 gene disruption in SRC-3 KO Tregs.
(A) Dual immunofluorescence for $Foxp3^{+}SRC\text{-}3^{+}$ cells in the spleens of SRC-$3^{f/f}$ and SRC-$3^{d/d}$:Treg female mice along with percentage calculations of Foxp3+SRC-3+ cells with respect to the total number of Foxp3+ cells. (B) Validation of isolated Tconv and Tregs from spleens of

SRC-3$^{f/f}$ and SRC-3$^{d/d}$:Treg female mice by flow cytometry with antibodies against CD25 and Foxp3. (C) Dual immunofluorescence for Foxp3^{+}SRC-3^{+} cells in CD4^{+}CD25^{+} Treg cells isolated from spleens of SRC-3$^{f/f}$ and SRC-3$^{d/d}$:Treg female mice along with percentage calculations of Foxp3^{+}SRC-3^{+} cells with respect to the total number of Foxp3+ cells. (D) Quantitative PCR (qPCR) for SRC-3 mRNA levels in SRC-3 KO Tregs. CD4^{+}CD25^{+}EGFP^{-} and CD4^{+}CD25^{+}EGFP^{+} T cells were isolated from the spleens of SRC-3$^{f/f}$ and SRC-3$^{d/d}$:Treg female mice. RNA was isolated from these T cells, and then SRC-3 mRNA was determined by qPCR with primers corresponding to exon 11 of the SRC-3 mRNA.

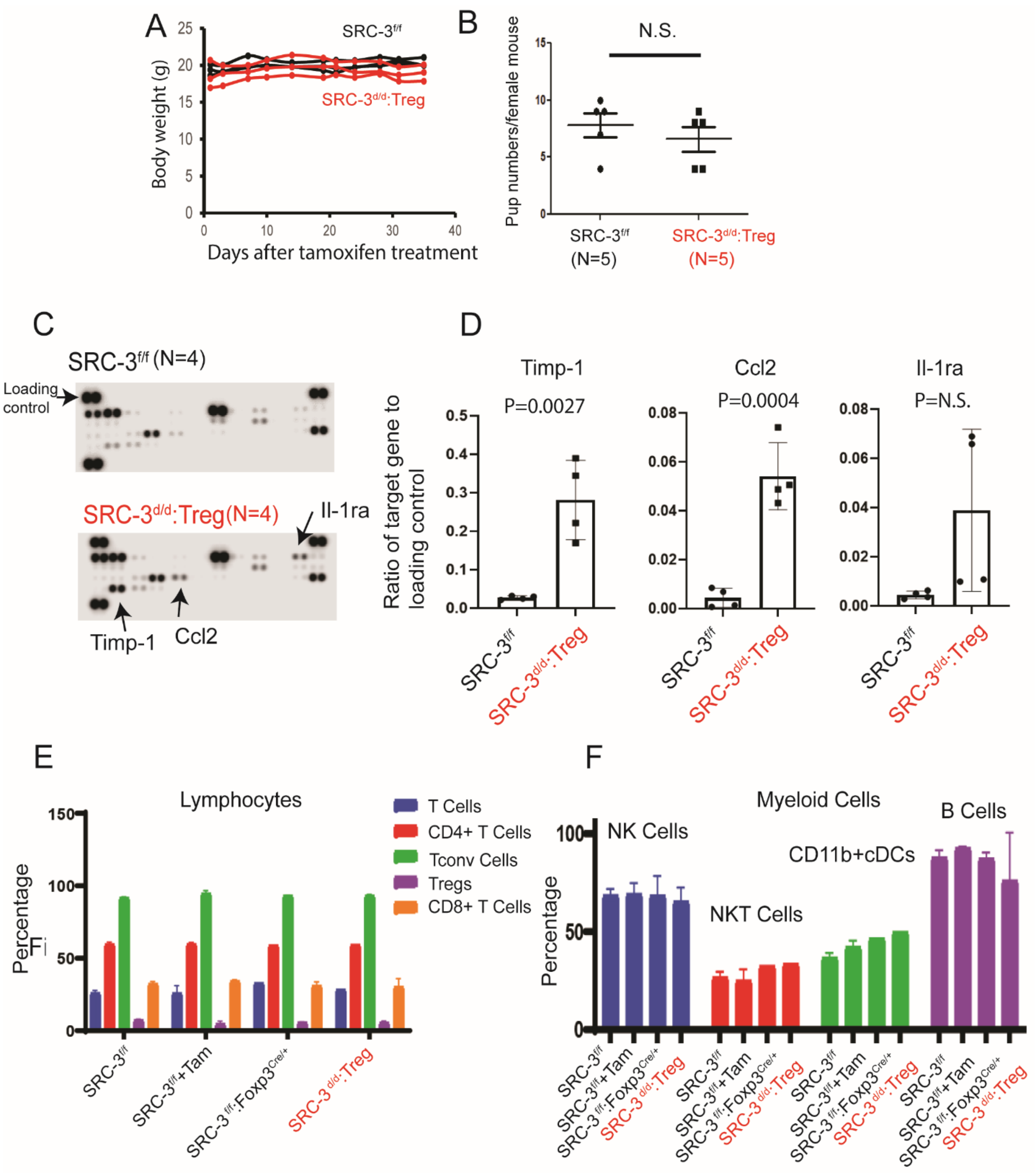

Fig. S4 Phenotype of SRC-3$^{d/d}$:Treg mice.

(A) Body weight measurements of SRC-3$^{f/f}$ and SRC-3$^{d/d}$:Treg female mice. These mice were treated with tamoxifen (five days) and then their weight was measured. (B) Fertility of SRC-3$^{f/f}$

versus SRC-3$^{d/d}$:Treg female mice. The number of pups born to SRC-3$^{f/f}$ and SRC-3$^{d/d}$:Treg female mice mated with wild-type male proven-breeders. (C) Cytokine profiles in blood from SRC-3$^{f/f}$ and SRC-3$^{d/d}$:Treg female mice. Blood was collected from these mice on the 30th day after tamoxifen treatment. (D) Quantification of Timp-1, Ccl2, and Il-1ra levels in the blood of SRC-3$^{f/f}$ and SRC-3$^{d/d}$:Treg female mice is shown in panel (C). (E-F) Immunophenotyping of lymphocytes (E) and myeloid cells (F) in spleens from SRC-3$^{f/f}$, tamoxifen-treated SRC-3$^{f/f}$, SRC-3$^{f/f}$:Foxp3$^{Cre-ERT2/+}$, and tamoxifen-treated SRC-3$^{f/f}$:Foxp3$^{Cre-ERT2/+}$ female mice. Spleens were harvested from these mice on the 30th day after either tamoxifen or vehicle treatment. NS, Non Specific.

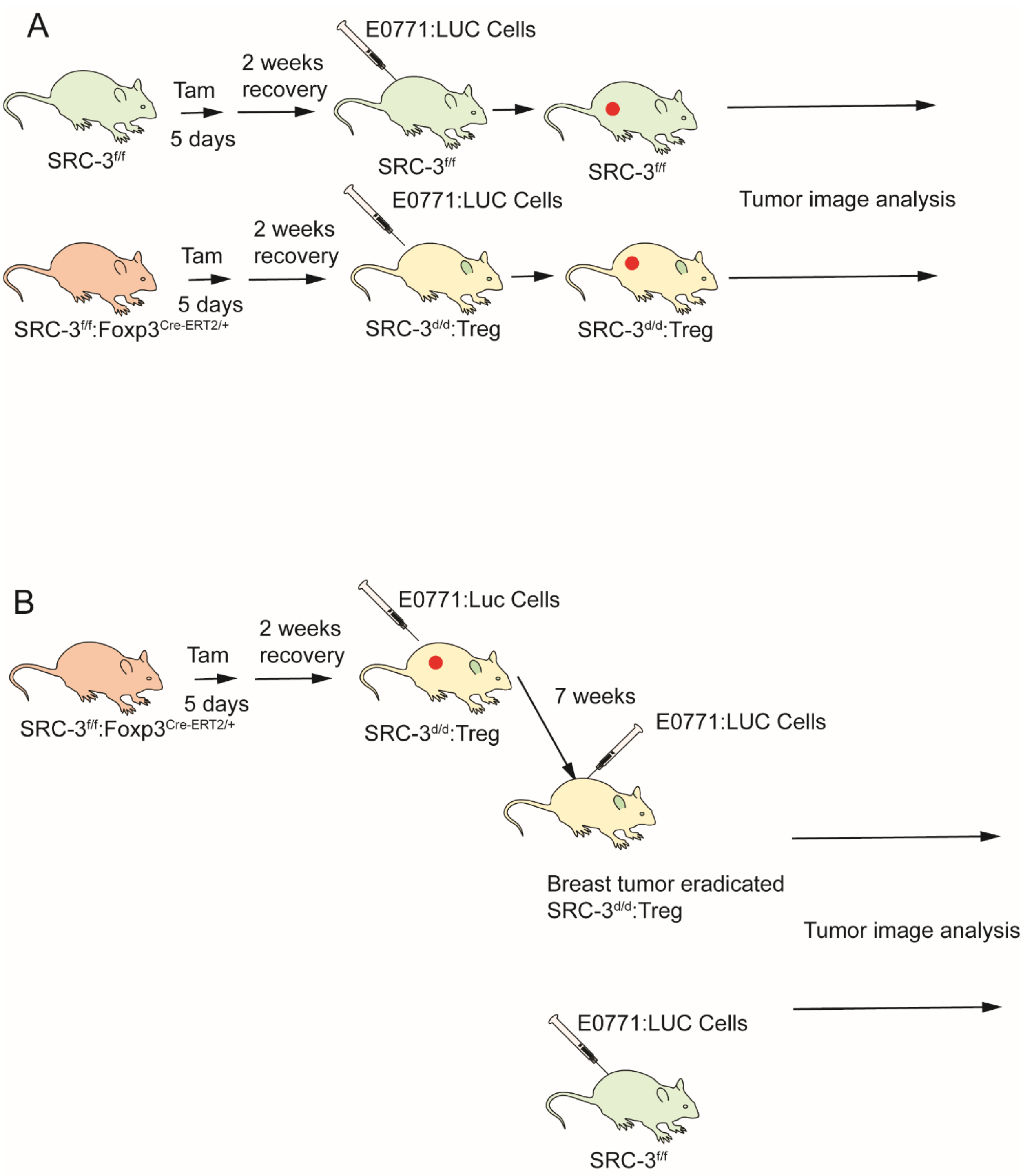

Fig. S5 Schematic diagram for SRC-3KO Treg mediated tumor eradication and resistance in SRC-3$^{d/d}$:Treg female mice.

(A) Diagram showing tumor eradication in SRC-3$^{d/d}$:Treg female mice. SRC-3$^{f/f}$ and SRC-3$^{f/f}$:Foxp3$^{Cre\text{-}ERT2}$ female mice were treated with tamoxifen (75 mg/kg for 5 days). After two

weeks, E0771:LUC cells ($1x10^5$ cells) were orthotopically injected into the mammary fat pad of SRC-$3^{f/f}$ and SRC-$3^{d/d}$:Treg female mice. Afterward, tumor luciferase activity images were collected. (B) Diagram defining tumor resistance in SRC-$3^{d/d}$:Treg female mice. E0771:LUC cells were orthotopically injected into SRC-$3^{d/d}$:Treg mice. After seven weeks, tumors were eradicated in SRC-$3^{d/d}$:Treg female mice, and then mice were orthotopically reinjected with E0771:LUC cells. As controls, E0771 cells were orthotopically injected into SRC-$3^{f/f}$ female mice.

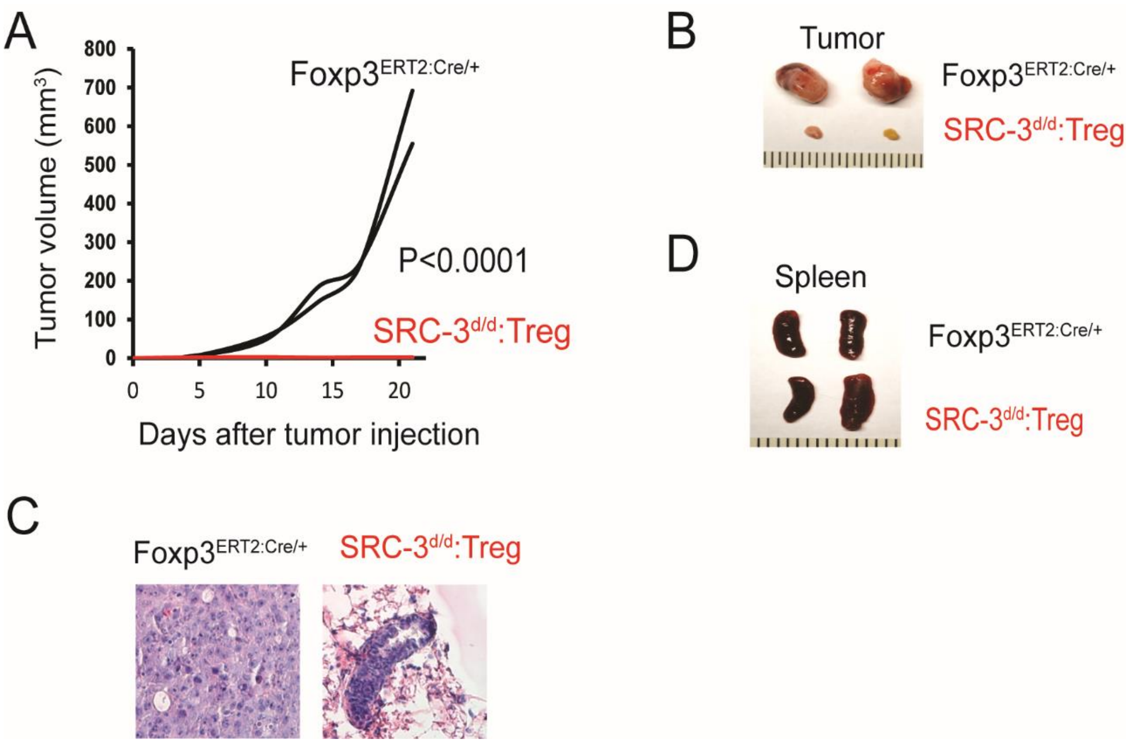

Fig. S6 Growth of E0771 breast tumors in Foxp3$^{Cre\text{-}ERT2}$ female mice.

(A) E0771 breast tumor growth in Foxp3$^{Cre\text{-}ERT2}$ and SRC-3$^{d/d}$:Treg female mice. (B) Breast tumors isolated from Foxp3$^{Cre\text{-}ERT2}$ and SRC-3$^{d/d}$:Treg female mice on the 23rd day after injection of E0771 cells. (C) H&E staining of tumors is presented in panel (B). (D) Spleens isolated from Foxp3$^{Cre\text{-}ER}$ and SRC-3$^{d/d}$:Teg female mice on the 40th day after tamoxifen treatment.

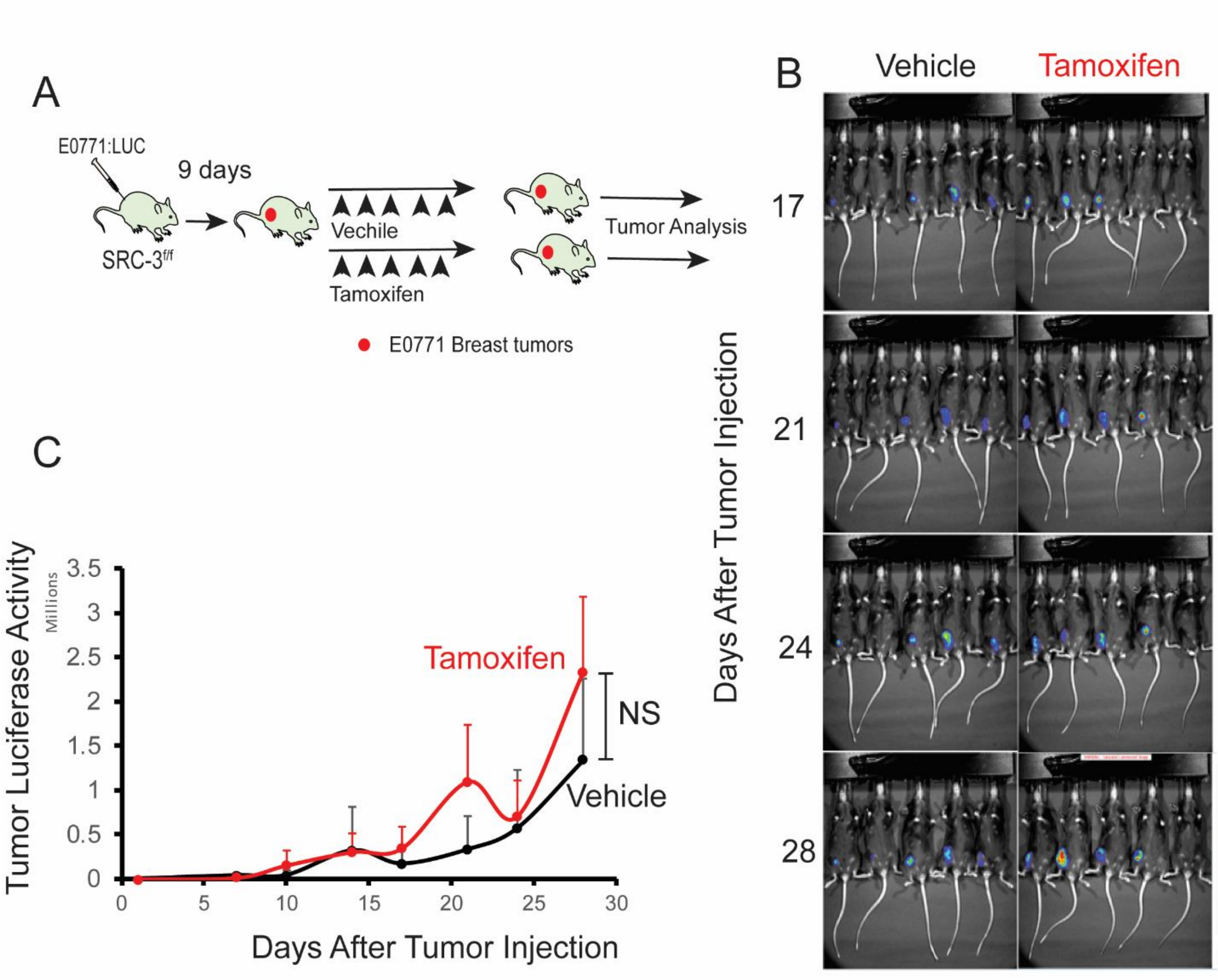

Fig. S7 Tamoxifen treatment did not impact the growth of E0771 breast tumors in SRC-3$^{f/f}$ female mice.

(A) Schematic describing tamoxifen treatment in tumor-bearing SRC-3$^{f/f}$ female mice. (B) Tumor luciferase activity of tumor-bearing SRC-3$^{f/f}$ female mice treated with tamoxifen versus vehicle. (C) The quantification of tumor luciferase activity in Panel B.

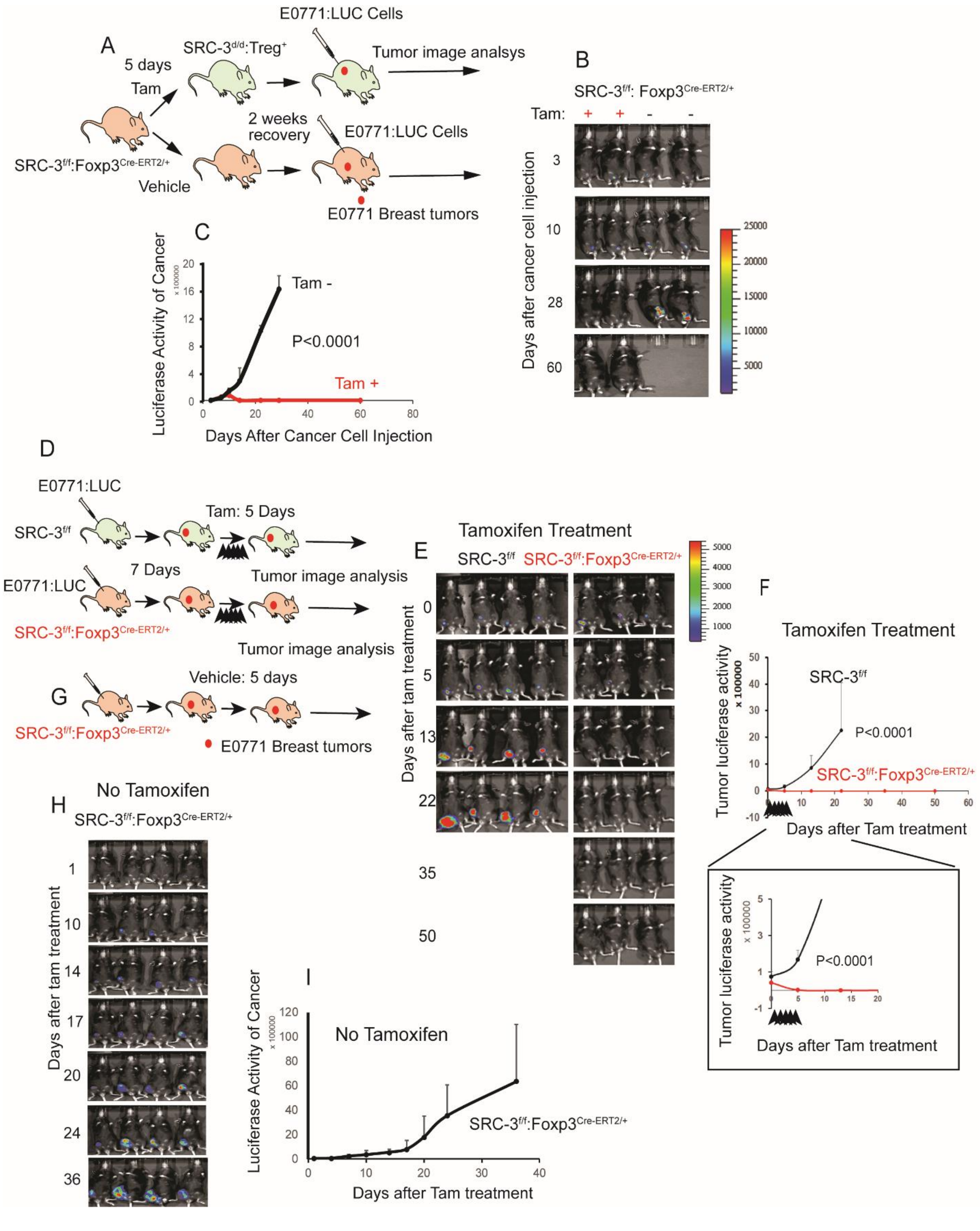

Fig. S8 Breast tumor regression and prevention in SRC-3$^{d/d}$:Treg female mice.

(A) Diagram to illustrate the suppression of tumor initiation by SRC-3 KO Treg cell induction. SRC-$3^{f/f}$:Foxp$3^{Cre\text{-}ERT2/+}$ female mice were treated with tamoxifen to generate SRC-$3^{d/d}$:Treg mice. As controls, SRC-$3^{f/f}$:Foxp$3^{Cre\text{-}ERT2/+}$ female mice were treated with vehicle instead. E0771 cells ($1x10^5$ cells) were orthotopically injected into SRC-$3^{f/f}$:Foxp$3^{Cre\text{-}ERT2/+}$ and SRC-$3^{d/d}$:Treg mice two weeks later after tamoxifen treatment. (B) Tumor luciferase activity images are presented in panel (A). (C) Quantification of the tumor luciferase images are presented in panel (B). (D) The diagram illustrates the tumor regression activity of SRC-3 KO Tregs. SRC-$3^{f/f}$ and SRC-$3^{f/f}$:Foxp$3^{Cre\text{-}ERT2/+}$ female mice were orthotopically injected with E0771 cells. After seven days, tumor-bearing SRC-$3^{f/f}$ and SRC-$3^{f/f}$:Foxp$3^{Cre\text{-}ERT2/+}$ mice were treated with tamoxifen for five days. Afterward, the luciferase activity emanating from tumors was determined. (E) Determination of luciferase activity from tumors is presented in panel (D). (F) Quantifying luciferase activity is presented in panel **e** until the ^{2}0th day, which is in the box, and the 50th day after tamoxifen treatment. (G) This diagram illustrates the tumor progression in SRC-$3^{f/f}$:Foxp$3^{Cre\text{-}ERT2/+}$ female mice upon vehicle treatment. (H) The tumor luciferase activity in panel G. (I) Quantitation of luciferase activity is presented in panel H. Tam, Tamoxifen.

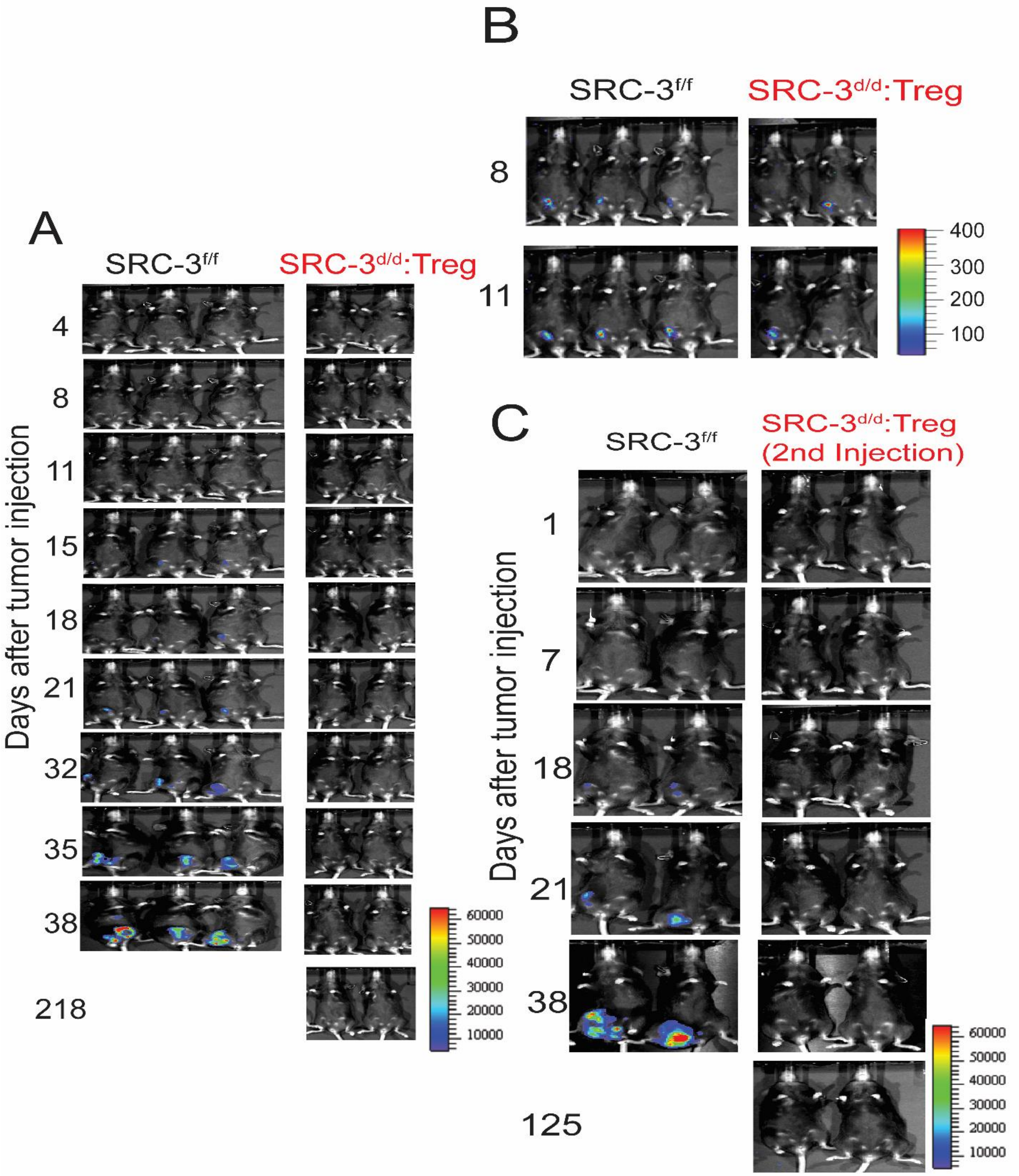

Fig. S9 E0771 breast tumor eradication in SRC-3$^{d/d}$:Treg mice.

(A) Breast tumor luciferase activity in SRC-3$^{f/f}$ and SRC-3$^{d/d}$:Treg female mice. (B) Tumor luciferase activity in tamoxifen-treated SRC-3$^{f/f}$ and SRC-3$^{d/d}$:Treg mice on the 8th and 11th days

after E0771 cell injection. (C) Tumor luciferase activity in SRC-$3^{f/f}$ and breast cancer-eradicated SRC-$3^{d/d}$:Treg mice after a 2nd injection of E0771:LUC cells.

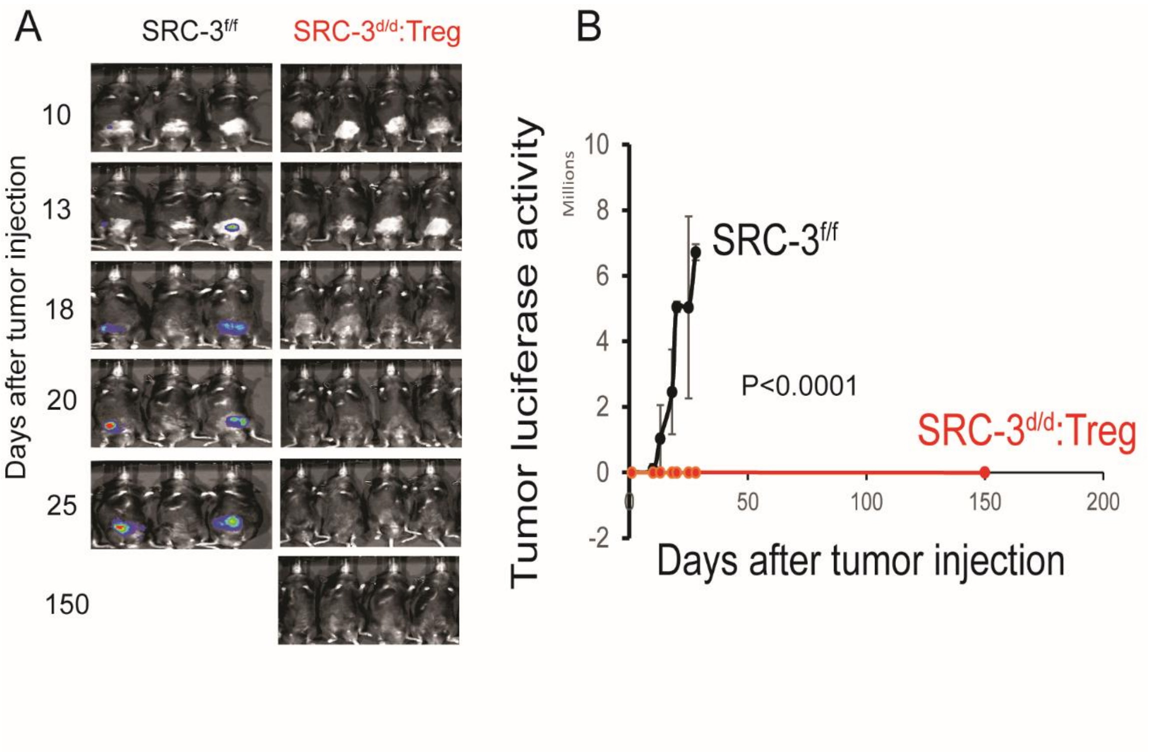

Fig. S10 Eradication of prostate cancer in SRC-3$^{d/d}$:Treg male mice.

A) Determination of the luciferase activity emanating from prostate tumors in SRC-3$^{f/f}$ and SRC-3$^{d/d}$:Treg male mice. Luciferase-labeled RM-1 mouse prostate cancer cells (1x10^{3} cells) were orthotopically injected into the prostate of SRC-3$^{f/f}$ and SRC-3$^{d/d}$:Treg male mice. Afterward, tumor luciferase activity arising from the prostate was determined. (B) Quantification of prostate tumor luciferase activity is presented in panel (A).

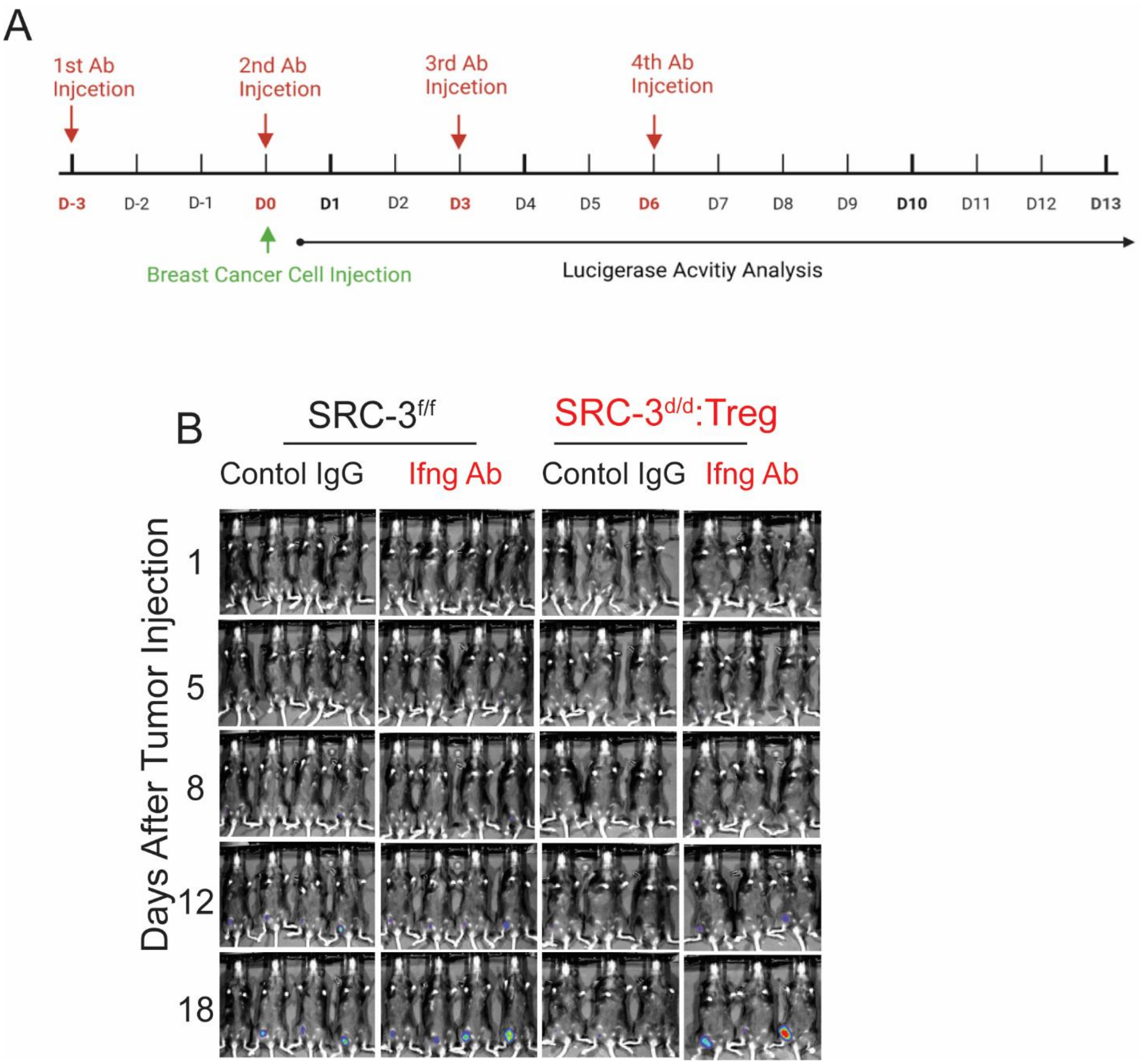

Fig. S11. Anti-Ifng antibody treatment prevents tumor eradication activity of SRC-3$^{d/d}$:Foxp3$^{Cre/+}$ Tregs in female mice.

(A) Schematic for anti-Ifng antibody and control rat IgG treatment of breast tumor-bearing SRC-3$^{f/f}$ and SRC-3$^{d/d}$:Treg mice. (B) Tumor luciferase activity of tumor-bearing SRC-3$^{f/f}$ and SRC-3$^{d/d}$:Treg female mice treated with anti-Ifng antibody (Ifng Ab) and control rat IgG (Control IgG).

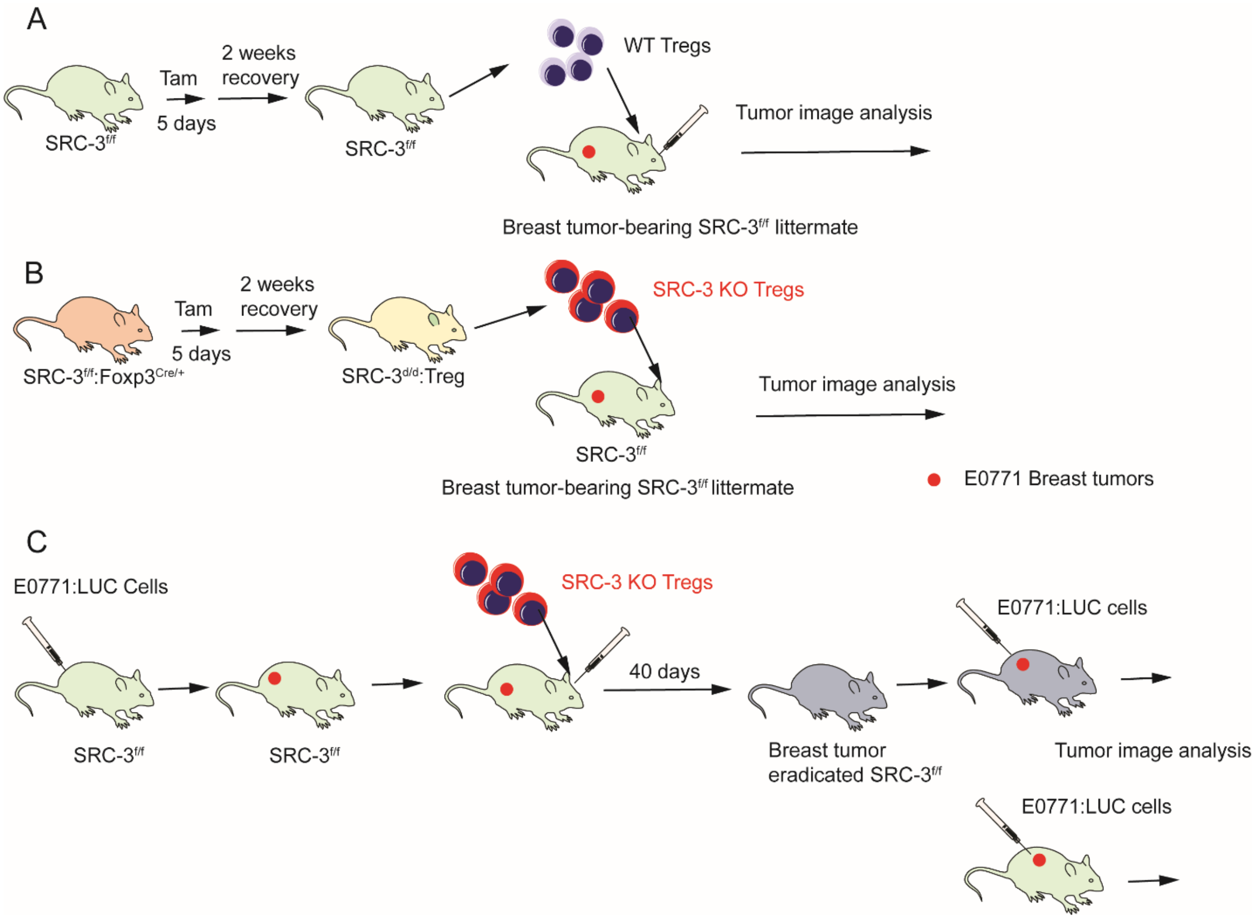

Fig. S12 Schematic diagram of the SRC-3KO Treg ACT process.

(A) ACT with wild-type Tregs was performed by isolating Tregs from the spleen of SRC-$3^{f/f}$ female mice treated with tamoxifen. These Tregs were then administrated into tumor-bearing SRC-$3^{f/f}$ female littermates by retro-orbital injection. (B) ACT with SRC-3 KO Tregs was performed similarly, except that SRC-$3^{d/d}$:Treg female mice were used as Treg donors. (C) Tumor-resistant mice generated after ACT with SRC-3 KO Tregs. SRC-3 KO Tregs were isolated from the spleens of SRC-$3^{d/d}$:Treg female mice. Tumor-bearing SRC-$3^{f/f}$ female mice were adoptively transferred with SRC-3 KO Tregs. Tumors were eradicated in these SRC-$3^{f/f}$ female mice by the 40th day after ACT. Afterward, E0771:LUC cells ($1x10^5$ cells) were orthotopically injected into tumor-eradicated SRC-$3^{f/f}$ female mice adoptively transferred with SRC-3 KO Tregs. As controls, E0771:LUC cells ($1x10^5$ cells) were orthotopically injected into SRC-$3^{f/f}$ female mice. Tam, Tamoxifen.

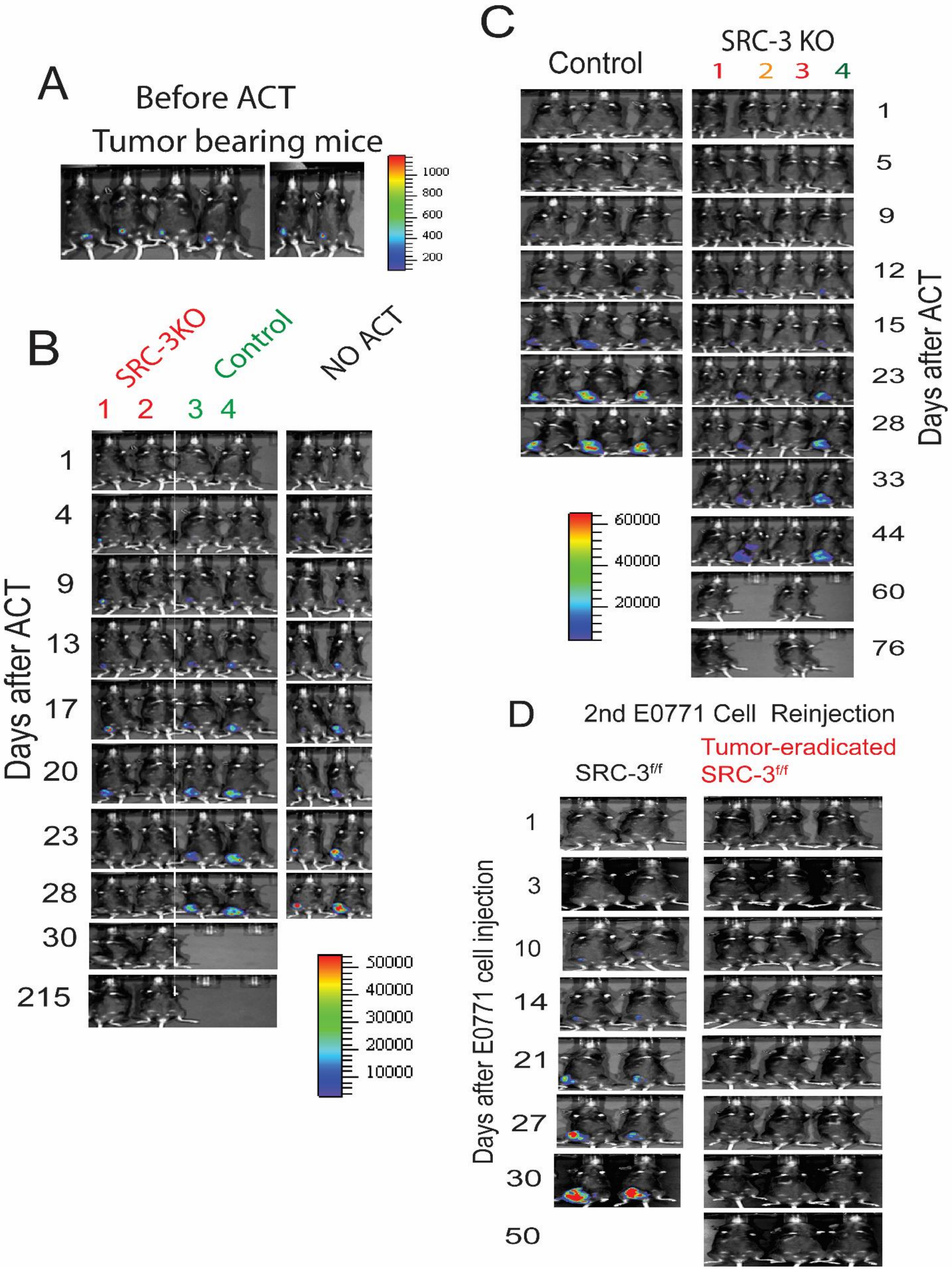
A
Before ACT
Tumor bearing mice
1000
800
600
400
200
B
SRC-3KO
Control
NO ACT
1 2 3 4
Days after ACT
1
4
9
13
17
20
23
28
30
215
50000
40000
30000
20000
10000
C
Control
SRC-3 KO
1 2 3 4
Days after ACT
1
5
9
12
15
23
28
33
44
60
76
60000
40000
20000
D
2nd E0771 Cell Reinjection
SRC-3f/f
Tumor-eradicated SRC-3f/f
Days after E0771 cell injection
1
3
10
14
21
27
30
50

Fig S13 Tumor eradication through adoptive SRC-3 KO Treg transfer.

(A) Tumor luciferase activity in tumor-bearing SRC-$3^{f/f}$ female mice before ACT. (B) Tumor luciferase image analysis in tumor-bearing SRC-$3^{f/f}$ female mice after ACT with SRC-3 KO Tregs (800K cells) and wild-type Tregs (800K cells). Control tumor-bearing SRC-$3^{f/f}$ mice that did not undergo ACT (NO ACT). (C) Tumor luciferase activity in SRC-$3^{f/f}$ female mice after ACT with different doses of SRC-3 KO Tregs. Tumor-bearing SRC-$3^{f/f}$ female mice were injected with adoptively transferred wild-type (800K cells) or SRC-3 KO Treg cells (1:610 K cells, 2:326 K cells, 3:643 K cells, 4:320 K cells). Afterward, the luciferase activity emanating from tumors was determined. (D) Tumor luciferase activity in tumor-eradicated SRC-$3^{f/f}$ female mice after ACT with SRC-3 KO Tregs and SRC-$3^{f/f}$ female control mice after a 2nd injection of E0771:LUC cells.

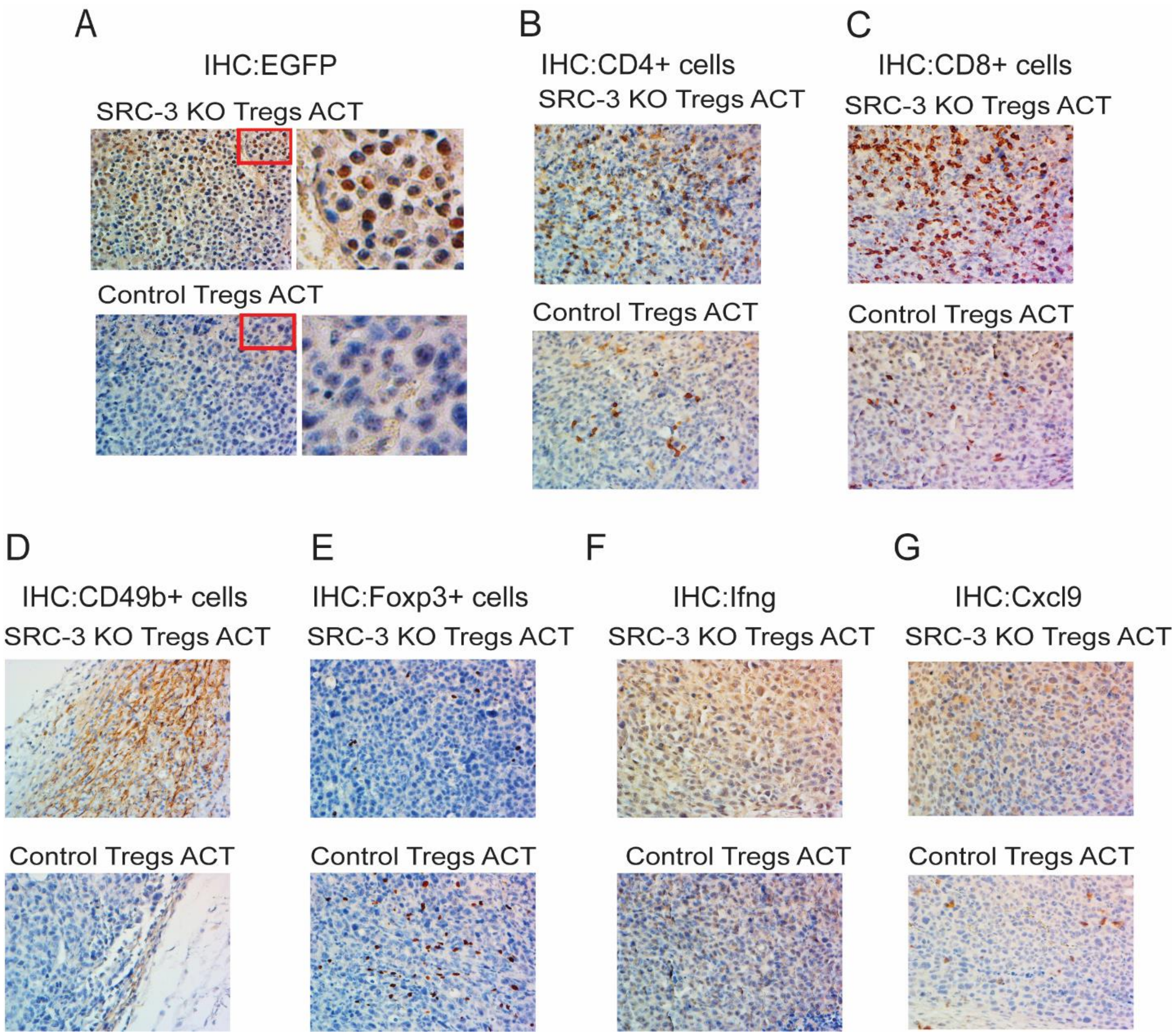

Fig. S14 Breast tumors acquire an antitumor immune environment after ACT with SRC-3 KO Tregs.

(A-G) IHC analysis of expression of EGFP (A), CD4+ (B), CD8+(C), CD49b+ NK cells (D), Foxp3 (E), Ifng (F) and Cxcl9 (G) in breast tumors in SRC-3$^{f/f}$ female mice receiving ACT with SRC-3 KO or WT control Tregs.

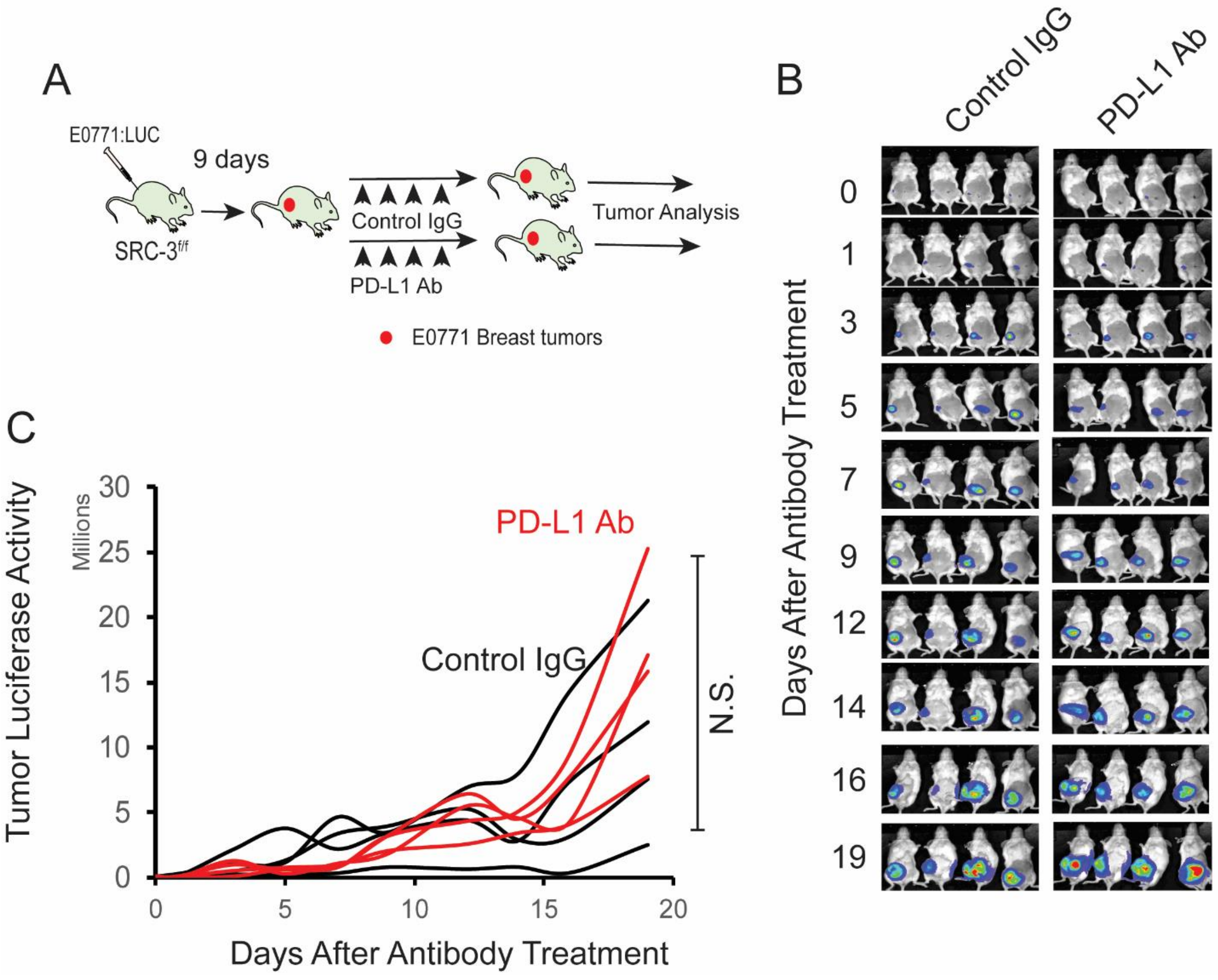

Fig. S15. Anti PD-L1 antibody did not suppress E0771 breast tumor progression in SRC-3$^{f/f}$ female mice.

(A) Schematic for anti-PD-L1 antibody treatment of breast tumor-bearing SRC-3$^{f/f}$ female mice.
(B) Tumor luciferase activity of tumor-bearing SRC-3 $^{f/f}$ female mice treated with anti-PD-L1 antibody (PD-L1 Ab, 100 μg/20g mouse) and control rat IgG (Control IgG, 100 μg/20g mouse).
(C) The quantification of tumor luciferase activity in Panel B.

Table S1 Pathways downregulated in SRC-3 KO Tregs

Pathway name	Entities pValue	Entities FDR	Submitted entities found
RUNX2 regulates genes involved in cell migration	7.05 E-04	0.48	Itga5;Runx2
Chemokine receptors bind chemokines	1.26 E-03	0.48	Cx3cl1;Ccl5;Cxcr3;Ccr9;Xcl1
Estrogen-dependent nuclear events downstream of ESR-membrane signaling	1.46 E-03	0.48	Fos;Elk1;Uhmk1;Hbegf
RUNX3 Regulates Immune Response and Cell Migration	2.93 E-03	0.58	Rorc;Runx3
Calcitonin-like ligand receptors	3.82 E-03	0.58	Calca
Erythropoietin activates Phospholipase C gamma (PLCG)	6.06 E-03	0.58	Lyn;Irs2;Plcg2
RUNX3 regulates BCL2L11 (BIM) transcription	1.25 E-02	0.58	Smad3;Runx3
RUNX2 regulates genes involved in differentiation of myeloid cells	1.25 E-02	0.58	Runx2
TGFBR1 KD Mutants in Cancer	1.25 E-02	0.58	Smad3;Tgfbr1
Regulation of KIT signaling	1.45 E-02	0.58	Lyn;Kit;Sos1
Erythropoietin activates RAS	1.67 E-02	0.58	Lyn;Irs2;Sos1
RUNX2 regulates chondrocyte maturation	1.68 E-02	0.58	Runx2
Loss of Function of TGFBR1 in Cancer	1.68 E-02	0.58	Smad3;Tgfbr1
SMAD2/3 Phosphorylation Motif Mutants in Cancer	1.68 E-02	0.58	Smad3;Tgfbr1
Signaling by Erythropoietin	1.75 E-02	0.58	Lyn;Irs2;Plcg2;Sos1
Signaling by Interleukins	1.94 E-02	0.58	Lyn;Il12rb1;Il13ra1;Cx3cl1;Cd80;Il17re;Rorc;Il18rap;Fscn1;Rora;Irs2;Fos;Cebpd;Elk1;Smad3;Il18r1;Ccl5;Anxa2;Sos1;Il9r
RUNX3 regulates CDKN1A transcription	2.15 E-02	0.58	Smad3;Runx3
Loss of Function of SMAD2/3 in Cancer	2.15 E-02	0.58	Smad3;Tgfbr1

GRB2 events in ERBB2 signaling	2.17 E-02	0.5 8	Nrg2;Sos1;Hbegf
SHC1 events in ERBB4 signaling	2.17 E-02	0.5 8	Nrg2;Sos1;Hbegf
Signaling by NOTCH2	2.28 E-02	0.5 8	Jag2;Gzmb;Cntn1
Erythropoietin activates STAT5	2.67 E-02	0.5 8	Lyn;Irs2
Signaling by TGF-beta Receptor Complex in Cancer	2.67 E-02	0.5 8	Smad3;Tgfbr1
Drug resistance of KIT mutants	2.75 E-02	0.5 8	Kit
KIT mutants bind TKIs	2.75 E-02	0.5 8	Kit
Regorafenib-resistant KIT mutants	2.75 E-02	0.5 8	Kit
Masitinib-resistant KIT mutants	2.75 E-02	0.5 8	Kit
Sorafenib-resistant KIT mutants	2.75 E-02	0.5 8	Kit
Imatinib-resistant KIT mutants	2.75 E-02	0.5 8	Kit
Nilotinib-resistant KIT mutants	2.75 E-02	0.5 8	Kit
Sunitinib-resistant KIT mutants	2.75 E-02	0.5 8	Kit
Dasatinib-resistant KIT mutants	2.75 E-02	0.5 8	Kit
NPAS4 regulates expression of target genes	2.90 E-02	0.5 8	Ret;Fos
Signaling by ERBB4	2.90 E-02	0.5 8	Nedd4;Nrg2;Adap1;Sos1;Hbegf
PI5P, PP2A and IER3 Regulate PI3K/AKT Signaling	3.03 E-02	0.5 8	Cd80;Pdgfrb;Kit;Nrg2;Irs2;Pip5k1b;Hbegf;Ie r3
Germ layer formation at gastrulation	3.05 E-02	0.5 8	Eomes;Smad3
RUNX3 regulates WNT signaling	3.24 E-02	0.5 8	Tcf4;Runx3
Binding of TCF/LEF:CTNNB1 to target gene promoters	3.24 E-02	0.5 8	Tcf4;Runx3
SOS-mediated signalling	3.24 E-02	0.5 8	Irs2;Sos1
SHOC2 M1731 mutant abolishes MRAS complex function	3.24 E-02	0.5 8	Braf;Shoc2

RUNX1 regulates estrogen receptor mediated transcription	3.24 E-02	0.5 8	Gpam
Interleukin-4 and Interleukin-13 signaling	3.72 E-02	0.5 8	Il13ra1;Rorc;Fscn1;Rora;Fos;Cebpd
APC-Cdc20 mediated degradation of Nek2A	3.73 E-02	0.5 8	Anapc10;Nek2;Bub1
Signaling by MRAS-complex mutants	3.85 E-02	0.5 8	Braf;Shoc2
Gain-of-function MRAS complexes activate RAF signaling	3.85 E-02	0.5 8	Braf;Shoc2
Interleukin-18 signaling	3.85 E-02	0.5 8	Il18r1;Il18rap
RUNX1 regulates transcription of genes involved in differentiation of myeloid cells	3.85 E-02	0.5 8	Runx2
Negative regulation of the PI3K/AKT network	4.07 E-02	0.5 8	Cd80;Pdgfrb;Kit;Nrg2;Irs2;Pip5k1b;Hbegf;Ie r3
FOXO-mediated transcription of cell cycle genes	4.09 E-02	0.5 8	Smad3;Klf4
Nuclear signaling by ERBB4	4.41 E-02	0.5 8	Nrg2;Adap1;Hbegf
Senescence-Associated Secretory Phenotype (SASP)	4.43 E-02	0.5 8	Anapc10;Fos;Igfbp7;Cebpb
Signaling by KIT in disease	4.47 E-02	0.5 8	Lyn;Kit;Sos1
Signaling by phosphorylated juxtamembrane, extracellular and kinase domain KIT mutants	4.47 E-02	0.5 8	Lyn;Kit;Sos1
Downregulation of TGF-beta receptor signaling	4.47 E-02	0.5 8	Smad3;Nedd4;Tgfbr1

Table S2 Pathways upregulated in SRC-3 KO Tregs

Pathway name	Entities pValue	Entities FDR	Submitted entities found
HDACs deacetylate histones	0.0012	0.38	Hdac1;H4c12;H3c11;H3c1;H4c1;Hist1h2al
p75NTR negatively regulates cell cycle via SC1	0.0026	0.38	Hdac1
Interferon alpha/beta signaling	0.0027	0.38	Stat1;Usp18;Isg15;Xaf1
ERCC6 (CSB) and EHMT2 (G9a) positively regulate rRNA expression	0.0032	0.38	Hdac1;H4c12;H3c11;H3c1;H4c1
Regulation of IFNA/IFNB signaling	0.0053	0.41	Stat1;Usp18
RHO GTPases activate KTN1	0.0100	0.41	Klc2
Formation of the beta-catenin:TCF transactivating complex	0.0101	0.41	Tle1;Hdac1;H4c12;H3c11;H3c1;H4c1
Repression of WNT target genes	0.0171	0.41	Tle1;Hdac1
Positive epigenetic regulation of rRNA expression	0.0181	0.41	Hdac1;H4c12;H3c11;H3c1;H4c1
NoRC negatively regulates rRNA expression	0.0181	0.41	Hdac1;H4c12;H3c11;H3c1;H4c1
Interleukin-6 signaling	0.0192	0.41	Stat1
ISG15 antiviral mechanism	0.0204	0.41	Stat1;Usp18;Isg15
STAT3 nuclear events downstream of ALK signaling	0.0214	0.41	Hdac1
RNA Polymerase I Promoter Clearance	0.0220	0.41	Hdac1;H4c12;H3c11;H3c1;H4c1
RNA Polymerase I Transcription	0.0237	0.41	Hdac1;H4c12;H3c11;H3c1;H4c1
Negative epigenetic regulation of rRNA expression	0.0255	0.41	Hdac1;H4c12;H3c11;H3c1;H4c1
Antiviral mechanism by IFN-stimulated genes	0.0303	0.41	Stat1;Usp18;Isg15
RMTs methylate histone arginines	0.0316	0.41	H4c12;H3c11;H3c1;H4c1;Hist1h2al
NOTCH1 Intracellular Domain Regulates Transcription	0.0346	0.41	Tle1;Hdac1
SUMOylation of chromatin organization proteins	0.0425	0.41	Hdac1;H4c12;H4c1

Transcriptional activity of SMAD2/SMAD3:SMAD4 heterotrimer	0.0460	0.41	Mapk7;Hdac1;Stat1
Notch-HLH transcription pathway	0.0477	0.41	Hdac1
Signaling by phosphorylated juxtamembrane, extracellular and kinase domain KIT mutants	0.0477	0.41	Stat1
Signaling by KIT in disease	0.0477	0.41	Stat1
Signalling to ERK5	0.0483	0.41	Mapk7

Abbreviations

SRC-3	Steroid Receptor coactivator-3
ACT	Adoptive cell transfer
Ccl	Chemokine (C-C motif) ligand
Ccr	Chemokine (C-C motif) Receptor
CTL	Cytotoxic T lymphocytes
Cxcl	C-X-C Motif Chemokine Ligand
CXCR	C-X-C chemokine receptor
EGFP	Enhanced green fluorescent protein
Foxp3	Forkhead box transcription factor 3
H&E	Hematoxylin and eosin
Ifng	Interferon-γ
IL	Interleukin
Il-1ra	Interleukin 1 receptor antagonist
IVIS	In Vivo Image System
Klrb1c	Killer cell lectin-like receptor subfamily B, member 1,
Klrk1	Killer Cell Lectin Like Receptor K1
KO	Knockout
LUC	Luciferase
Mip	Macrophage Inflammatory Proteins
NS.	Nonspecific
NK.	Natural Killer
PLC	Percentage of Labelled Cells
TCR	T Cell Receptor
TGF-β	Transforming growth factor beta
Tigit	T Cell Immunoreceptor With Ig And ITIM Domains
Timp-1	Tissue inhibitor matrix metalloproteinase 1
Treg	Regulatory T cells

Reference

1. Liu Z, Liao L, Zhou S, & Xu J (2008) Generation and validation of a mouse line with a floxed SRC-3/AIB1 allele for conditional knockout. *International journal of biological sciences* 4(4):202-207.

2. Zhang GL, Zhang Y, Cao KX, & Wang XM (2019) Orthotopic Injection of Breast Cancer Cells into the Mice Mammary Fat Pad. *Journal of visualized experiments : JoVE* (143).

3. Schindelin J, Rueden CT, Hiner MC, & Eliceiri KW (2015) The ImageJ ecosystem: An open platform for biomedical image analysis. *Molecular reproduction and development* 82(7-8):518-529.

4. Pavese J, Ogden IM, & Bergan RC (2013) An orthotopic murine model of human prostate cancer metastasis. *J Vis Exp* (79):e50873-e50873.

5. Gao M, *et al.* (2020) Therapy With Carboplatin and Anti-PD-1 Antibodies Before Surgery Demonstrates Sustainable Anti-Tumor Effects for Secondary Cancers in Mice With Triple-Negative Breast Cancer. *Front Immunol* 11:366.

6. Bankhead P, *et al.* (2017) QuPath: Open source software for digital pathology image analysis. *Sci Rep* 7(1):16878.
